# Integrated Metabolic and Immune Molecular Subtyping and a Molecular Classification Score for Revealing Disease Heterogeneity in Pulmonary Arterial Hypertension

**DOI:** 10.3390/metabo16090637

**Published:** 2026-09-01

**Authors:** Xin Chen, Yuetong Zhu, Qingping Shi, Jianing He, Siyu Chen, Jieru Han

**Affiliations:** 1 First Clinical Medical College, Heilongjiang University of Chinese Medicine, Harbin 150040, China; radinen@outlook.com (X.C.); 18646078578@163.com (J.H.); guanzirmer@outlook.com (S.C.); 2 School of Basic Medical Sciences, Heilongjiang University of Chinese Medicine, Harbin 150040, China; suiyuehuqibuyanxi@163.com (Y.Z.); sqp17326705862@163.com (Q.S.)

**Keywords:** pulmonary arterial hypertension, metabolic-immune crosstalk, molecular subtyping, risk score, transcriptomics, immune microenvironment, drug repositioning

## Abstract

Objectives: Despite the central role of metabolic–immune crosstalk in pulmonary arterial hypertension (PAH), integrative quantitative tools remain lacking. We aimed to construct and validate a metabolic–immune molecular classification score (MIRS) as a quantitative metric to capture the inflammation–metabolism balance and to assess its discriminatory performance for molecular subtyping, cross-cohort applicability, and biological implications. Methods: We integrated five PAH lung tissue transcriptomic datasets from the Gene Expression Omnibus (GEO). A training set was used to identify differentially expressed genes and to derive MIRS as the first principal component of 20 core genes. MIRS was projected onto independent validation cohorts. Immune microenvironment, pathway activities, protein–protein interaction, and drug repositioning analyses were performed. Results: MIRS significantly distinguished Non-IPAH from IPAH in GSE117261 (AUC = 0.718, *p* = 0.010) and revealed internal heterogeneity in SSc-PAH. MIRS-related gene expression patterns showed significant differences across COPD and ILD lung tissues in independent datasets. A unified fixed PCA projection pipeline with mean imputation for missing core genes was applied to all pulmonary disease datasets, enabling consistent numerical quantification of MIRS across cohorts under the same mathematical framework. MIRS failed to discriminate PAH from controls in PBMCs, indicating that this tissue-derived signature is not readily detectable in peripheral blood—a limitation that restricts its applicability to lung tissue specimens and highlights challenges for blood-based biomarker development in PAH. Higher MIRS correlated with increased immune scores and myeloid cell infiltration in Non-IPAH. Drug prediction identified sirolimus and tocilizumab as top candidates, with MIRS-stratified sensitivity patterns aligning with pathway loading directions. Conclusions: MIRS is a quantitative, tissue-restricted metric that captures metabolic–immune activation in PAH, with exploratory observations in other pulmonary diseases that warrant further validation. It provides a molecular stratification basis for subtype discrimination and a hypothesis-generating framework for future therapeutic investigations.

## 1. Introduction

Pulmonary arterial hypertension (PAH) is a rare cardiovascular disorder characterized by a progressive increase in pulmonary vascular resistance. The pathological core of the disease involves remodeling of the small pulmonary arteries, ultimately leading to right heart failure and death [1,2]. Despite substantial advances in targeted therapies over recent years, the 5-year survival rate of PAH patients remains only 60–70% [1]. The clinical heterogeneity of PAH suggests the existence of complex underlying molecular mechanisms, underscoring the urgent need for molecular stratification tools capable of capturing the biological essence of the disease.

The pathogenesis of PAH involves two core processes: metabolic reprogramming and inflammatory–immune activation. At the metabolic level, pulmonary vascular cells in PAH exhibit a pronounced “Warburg effect”—a preferential reliance on glycolysis for energy production even under aerobic conditions—accompanied by mitochondrial dysfunction and enhanced ribosome biogenesis [3,4,5]. These metabolic alterations provide the energy and biosynthetic basis for excessive vascular cell proliferation. At the inflammatory–immune level, there is evident inflammatory cell infiltration in the lung tissues of PAH patients, along with significantly elevated levels of pro-inflammatory cytokines and sustained activation of the NF-κB signaling pathway [6,7]. Notably, metabolic reprogramming and inflammatory activation are not independent events; metabolic intermediates can directly modulate immune cell function, while inflammatory signals can reciprocally drive metabolic pathway reprogramming [8]. This metabolic–immune interplay constitutes an important dimension of PAH pathobiology; however, effective clinical quantitative tools are currently lacking.

In recent years, transcriptomics-based molecular subtyping studies have provided new insights for precision medicine in PAH. Several studies have utilized transcriptomic data from lung tissues or blood samples to stratify PAH patients into distinct molecular subtypes [9]. For instance, one study used unsupervised clustering to divide idiopathic PAH (IPAH) patients into subgroups characterized by either inflammatory signals or metabolic alterations, and these subtypes differed in prognosis and treatment response [9]. However, existing molecular subtyping approaches have mostly focused on a single disease subtype or a single biological dimension and lack a quantitative method that integrates metabolism and immunity into a unified framework. Moreover, the degree of molecular overlap between PAH and other pulmonary diseases remains unclear.

On this basis, the present study aimed to construct a metabolic–immune risk score (MIRS) using multi-cohort PAH lung tissue transcriptomic data. MIRS was defined as the first principal component of a principal component analysis (PCA) based on 20 core genes (10 inflammation-related and 10 ribosome/metabolism-related genes) to quantify the balance between inflammation and metabolism in PAH lung tissues.

## 2. Materials and Methods

### 2.1. Data Sources and Preprocessing

Five human lung tissue PAH transcriptomic microarray datasets were obtained from the Gene Expression Omnibus (GEO) public database [10]: GSE53408 [11], GSE113439 [12], GSE15197 [13] (combined as the training set), GSE117261 [9] (internal independent validation set), and GSE48149 [14] (external SSc-PAH validation set). The platform, sample information, clinical subtypes, and intended use of each dataset are summarized in Table 1.

The training set comprised 44 PAH lung tissue samples after merging GSE53408 (*n* = 12), GSE113439 (*n* = 14, with one WHO group 4 CTEPH sample excluded due to fundamental mechanistic differences), and GSE15197 (*n* = 18). The internal validation set GSE117261 originally contained 83 samples, but 25 Failed Donor samples with tissue damage were excluded, leaving 58 valid PAH cases (32 IPAH and 26 Non-IPAH). The external validation set GSE48149 included 18 SSc-PAH samples, further divided into primary pulmonary hypertension (PPH) (*n* = 8) and SSc (*n* = 10) subgroups.

#### Data Standardization and Batch Correction

Affymetrix arrays were Robust Multi-array Average normalized (RMA-normalized), while Agilent and Illumina arrays were quantile-normalized, and all expression profiles were log_2_-transformed. Probe-to-gene mapping was performed using the official platform annotation files, and multiple probes for the same gene were collapsed by taking the mean expression value. Low-expression genes were filtered out if their expression was below baseline in more than 50% of samples, retaining 18,630 protein-coding genes for subsequent analyses.

To account for cross-platform technical variation, we adopted a two-pronged strategy appropriate for different analytical steps. For unsupervised visualization, we applied the removeBatchEffect function from the limma package (v3.50.0) to adjust the expression data, with dataset ID as the batch variable. For differential expression analysis and all subsequent statistical modeling, we did not pre-correct the data. Instead, we included dataset ID as a covariate in the linear model design matrix (design~0 + group + dataset_id). This approach preserves residual degrees of freedom and avoids the risk of inflated false-positive rates associated with using batch-corrected data for hypothesis testing. The effectiveness of batch adjustment for visualization was assessed by PCA of the full gene expression matrix before and after removeBatchEffect adjustment, judging by the degree of clustering of samples from different datasets.

### 2.2. Differential Expression Analysis and Construction of the MIRS Metabolic–Immune Risk Score

#### 2.2.1. Unsupervised Clustering and Differential expreSSIon Analysis

To identify genes with sufficient variation for differential expression analysis, we computed the standard deviation of expression across all genes in the 44 training samples and retained the top 2000 most variable genes. Differential expression analysis between IPAH and Non-IPAH clinical phenotypes was performed using limma (v3.50.0) with a linear model and eBayes empirical Bayes shrinkage; DEGs were defined as genes with |log_2_FC| > 0.5 and Benjamini–Hochberg adjusted *p* < 0.05.

#### 2.2.2. Pathway Enrichment and Core Gene Selection

To identify the core genes for MIRS, we implemented a multi-step selection pipeline that strictly separates pathway discovery from gene prioritization.

Step 1—Pathway discovery (clinical labels used for biological context only): We performed differential expression analysis between IPAH and Non-IPAH within the training set using limma (|log_2_FC| > 0.5, adj. *p* < 0.05). KEGG enrichment analysis of the resulting DEGs (clusterProfiler v4.2.0, unadjusted *p* < 0.05) revealed significant enrichment in three pathways central to PAH pathobiology: NF-κB inflammatory signaling (hsa04064), ribosome biogenesis (hsa03008), and mTORC1 signaling (hsa04150). This step was performed solely to identify relevant pathway categories—not to select the final 20 genes.

Step 2—Objective gene prioritization within pathways (clinical labels used only as a quantitative metric, not as a selection filter): From the full set of genes annotated to the three pathways identified in Step 1, we applied the following pre-specified, quantitative criteria to select the final 20 genes:

  (i) Genes were required to be among the top 2000 most variable genes in the training set (based on expression standard deviation), ensuring sufficient inter-sample variation;
 (ii) Genes were required to have |log2FC| > 1.0 between IPAH and Non-IPAH;
(iii) Genes were required to have Benjamini–Hochberg adjusted *p* < 0.01 in the IPAH vs. Non-IPAH comparison.

Among genes meeting all three criteria, we selected the top 10 genes with the largest |log_2_FC| from the NF-κB pathway as the inflammation-related set, and the top 10 genes with the largest |log_2_FC| from the combined ribosome/mTOR pathways as the ribosome/metabolism-related set. This ranking-based, effect-size-driven approach yielded the final 20-gene signature: inflammation-related genes—NFKB1, NFKB2, ICAM1, RELB, IL6, CXCL8, RELA, SELE, REL, IL1B; ribosome/metabolism-related genes—RICTOR, DEPTOR, RPS3A, RPL7, RPL35, RPS11, RPL11, RPL31, RPS6KB, TSC1.

The IPAH vs. Non-IPAH labels were used to quantify effect sizes (|log_2_FC|) for ranking genes, but were not used to construct the PCA model defining MIRS, which was performed without clinical labels. The labels were reserved for the independent validation stage (GSE117261).

To assess the robustness of this 20-gene signature against overfitting, we performed a leave-one-gene-out (LOGO) sensitivity analysis within the training set: each of the 20 genes was sequentially removed, PCA was retrained on the remaining 19 genes, and MIRS was recalculated for all samples. The resulting 19-gene MIRS values were correlated with the original 20-gene MIRS. The median Spearman correlation across all 20 iterations was ρ = 0.97 (range: 0.93–0.99) (Appendix C), confirming that no single gene dominates the signature and that the overall MIRS signal is collectively driven by the entire gene panel.

#### 2.2.3. PCA Definition of MIRS

Principal component analysis (PCA) was performed on the expression matrix of these 20 genes in the training set using prcomp (center = TRUE, scale = TRUE). Principal Component 1 (PC1) explained 41.4% of the total variance and was defined as the metabolic–immune risk score (MIRS). To evaluate the stability of the PC1 loading vector, we performed 1000 bootstrap resampling iterations on the training set. For each bootstrap sample, PCA was re-run and PC1 loadings were recalculated. The 95% confidence intervals (percentile method) for each of the 20 gene loadings were derived from the bootstrap distribution. All inflammation-related genes maintained consistently positive loadings and all ribosome/metabolism-related genes maintained consistently negative loadings across all bootstrap samples, confirming the directional stability of the MIRS signature. PC1 was manually oriented so that higher MIRS values represent relatively activated inflammatory signaling and suppressed ribosomal/metabolic signaling. To exclude the possibility that MIRS primarily reflects non-biological confounders, we performed platform effect assessment, sex association analysis, and Fisher exact test for non-random loading separation. Full details are provided in Appendix B.

The definition of MIRS and its PC1 loading vector were derived exclusively from the training set (*n* = 44) without any use of validation cohort data. All subsequent performance evaluations—including discrimination of IPAH from Non-IPAH, SSc-PAH heterogeneity analysis, cross-disease applicability in /COPD/ILD, and tissue specificity assessment in PBMC—were conducted in entirely independent cohorts that had no role in feature selection, PCA loading calculation, or parameter tuning. This strict separation of model development and validation phases minimizes the risk of overfitting.

Because the signs of PCA eigenvectors are mathematically arbitrary, we manually oriented PC1 to facilitate biological interpretation. Specifically, the PC1 loading vector and all PC1 scores were multiplied by −1 simultaneously—a transformation that preserves all PCA properties including variance explained, orthogonality, and relative sample distances—so that inflammation-related genes (NFKB1, NFKB2, ICAM1, RELB, IL6, CXCL8, RELA, SELE, REL, IL1B) carried positive loadings and ribosome/metabolism-related genes (RICTOR, DEPTOR, RPS3A, RPL7, RPL35, RPS11, RPL11, RPL31, RPS6KB, TSC1) carried negative loadings. This orientation decision was made exclusively on the training set and was fixed prior to any analysis of the validation cohorts, ensuring that no validation set information influenced the definition of MIRS. The loadings before and after orientation, along with 95% bootstrap confidence intervals, are provided in Supplementary Table S2.

#### 2.2.4. Cross-Cohort Fixed Projection for MIRS Calculation

To ensure consistent numerical quantification of MIRS across datasets, all cohorts included in this study were processed under one of two projection strategies, depending on whether the microarray platform fully covered the 20 core MIRS genes.

Strategy 1—Fixed 20-gene projection (primary analysis): This strategy was applied to all PAH cohorts (GSE117261, GSE48149) and to the COPD/ILD dataset (GSE47460). These datasets retained full coverage of all 20 core MIRS genes on their respective microarray platforms. No missing value imputation was required or performed. MIRS was calculated as MIRS = X·W, where W is the PC1 loading vector derived exclusively from the training set, with centering and scaling parameters fixed to the training set values. This strategy produces numerically comparable MIRS values across all cohorts to which it is applied.

Strategy 2—Shared-gene projection (exploratory PBMC analysis only): This strategy was used exclusively for the PBMC dataset (GSE33463), where the platform lacked full coverage of the 20 core genes. Under this strategy, PCA was retrained on the overlapping gene set between the training set and the PBMC platform—i.e., only genes present on both platforms were retained. MIRS values generated under Strategy 2 are mathematically distinct from and NOT numerically comparable to those from Strategy 1. Therefore, PBMC MIRS values were used exclusively for within-dataset group comparisons (PAH vs. Control) based on Wilcoxon rank-sum test *p*-values and ROC AUC, rather than for cross-dataset numerical comparisons. No mean imputation or any other form of missing value replacement was applied to any dataset under either strategy.

#### 2.2.5. Distinction Between Fixed-Projection and Shared-Gene Projection Strategies

Two mathematically distinct PCA projection strategies were applied:

(1) Fixed 20-gene projection (strategy1): Applied to all PAH cohorts (GSE117261, GSE48149) and to GSE47460 (COPD/ILD). These datasets retained full coverage of the 20 core MIRS genes. MIRS was calculated as MIRS = X·W, where W is the PC1 loading vector derived exclusively from the training set, with centering and scaling parameters fixed to the training set values. This strategy produces numerically comparable MIRS values across all cohorts to which it is applied.
(2) Shared-gene projection (Strategy 2): Used only for the PBMC dataset (GSE33463), where the platform lacked full coverage of the 20 core genes. Under this strategy, PCA was retrained on the overlapping gene set between the training set and the PBMC platform. MIRS values from Strategy 2 are mathematically distinct from and NOT numerically comparable to those from Strategy 1. Therefore, PBMC MIRS values were used exclusively for within-dataset group comparisons (PAH vs. Control) based on Wilcoxon rank-sum test *p*-values and ROC AUC, rather than for cross-dataset numerical comparisons.

All primary conclusions of this study—the discriminatory performance of MIRS in distinguishing IPAH from Non-IPAH (AUC = 0.718) and the characterization of SSc-PAH heterogeneity—are derived solely from Strategy 1 cohorts with complete 20-gene coverage and mathematically identical MIRS calculations.

#### 2.2.6. Quantitative Assessment of Cross-Platform and Cross-Cohort Comparability

To provide quantitative evidence that the fixed PCA projection strategy yields comparable MIRS values across independent cohorts despite differences in microarray platforms, we performed three complementary analyses:

First, we assessed cross-platform gene expression correlation for the 20 MIRS core genes. Using the 18 overlapping PAH samples from the training set that were profiled on both the Affymetrix GPL6244 (GSE53408/GSE113439) and Agilent GPL6480 (GSE15197) platforms, we calculated the Spearman correlation coefficient for each of the 20 core genes between platforms. The median correlation across all 20 genes was ρ = 0.87 (range: 0.72–0.95), confirming that the expression patterns of the MIRS core genes are largely preserved across the two major platform types used in this study.

Second, we compared the PC1 loading vectors between cohorts. For each independent PAH validation cohort (GSE117261, GSE48149), we extracted the 20-gene expression matrix, performed PCA independently, and calculated the cosine similarity between the resulting PC1 loading vector and the fixed training-set loading vector. The cosine similarity ranged from 0.89 to 0.96 (Table S1), indicating that the direction of the inflammation–metabolism axis is consistent across datasets despite differences in sample composition and platform.

Third, we performed a platform effect distance analysis. Three samples from the GSE53408 dataset (GPL6244 platform) were projected onto the training set PCA space. The mean Euclidean distance of these samples to the training set centroid was 1.24 ± 0.31, which fell within the 95% confidence interval of the training set inter-sample distances (0.89–1.67). This confirms that platform-related technical variation does not substantially distort the MIRS projection space, and that the fixed PCA model effectively segregates technical noise into principal components orthogonal to PC1 (MIRS).

These analyses collectively demonstrate that, under Strategy 1, MIRS numerical values are comparable across independent cohorts despite differences in platform, sample source, and preprocessing history.

### 2.3. Validation in Internal and External Cohorts

For the internal validation set GSE117261, MIRS was calculated for all 58 valid samples using the fixed projection rule. Samples were stratified by clinical phenotype into IPAH (*n* = 32) and Non-IPAH (*n* = 26). The difference in MIRS between the two groups was assessed by two-sided Mann–Whitney Wilcoxon rank-sum test, and the discriminatory performance was evaluated by receiver operating characteristic (ROC) curve analysis using the Package for Receiver Operating Characteristic Curves (pROC) package (v1.18.0) to compute the area under the curve (AUC) and its 95% confidence interval. For the external SSc-PAH cohort GSE48149, MIRS was similarly computed for the 18 samples, and the PPH (*n* = 8) and SSc (*n* = 10) subgroups were compared using the Wilcoxon rank-sum test.

### 2.4. Quantitative Assessment of the Lung Tissue Immune Microenvironment

The Estimation of Stromal and Immune cells in Malignant Tumor tissues using Expression data (ESTIMATE) algorithm [15] (v1.0.13) was applied to the batch-corrected standardized expression matrix to calculate immune scores, stromal scores, and combined ESTIMATE scores for each sample. Spearman rank correlation was then used to assess the association between MIRS and these scores, stratified by IPAH and Non-IPAH groups. In addition, single-sample gene set enrichment analysis (ssGSEA) from the Gene Set Variation Analysis (GSVA) package was performed using the 16 immune cell type signature gene sets published by Bindea et al. [16] to estimate the infiltration abundance of each immune cell type. Differential abundance between IPAH and Non-IPAH was tested with limma, and BH-adjusted *p* < 0.05 was considered significant.

### 2.5. Hallmark Metabolic and Immune Pathway Activity Analysis

Twenty-two Hallmark gene sets related to metabolism and immunity were downloaded from the MSigDB database [17]. In the GSE117261 validation set, ssGSEA was used to calculate pathway enrichment scores via GSVA (v1.42.0), and all scores were Z-score standardized. Differential pathway activity between IPAH and Non-IPAH was compared using limma, and Spearman correlation was used to examine the relationship between pathway scores and MIRS.

### 2.6. Quartile-Based Trend Tests for MIRS Stratification

All valid samples from GSE117261 were divided into quartiles based on their MIRS values (Q1, Q2–Q3, Q4). Because the Q4 group contained only 3 samples with insufficient statistical power, Q2–Q4 were merged into a single group, yielding three ordered groups: Group 1 (Q1, *n* = 37), Group 2 (Q2–Q3, *n* = 43), and Group 3 (Q4, *n* = 3). The trend of the binary outcome (proportion of Non-IPAH) across increasing MIRS was tested by the Cochran–Armitage trend test, and the ordered trend of the continuous outcome (immune score) was assessed by the Jonckheere–Terpstra test using the clinfun package (v1.1.2). All trend tests were one-sided with α = 0.05.

### 2.7. Protein–Protein Interaction (PPI) Network Analysis

A set of 100 MIRS-related feature genes was defined by taking the 20 core MIRS genes plus the top 80 genes that exhibited co-expression associations with the core genes among all 3268 DEGs from the training set. This gene set was used for PPI network construction using the Search Tool for the Retrieval of Interacting Genes/Proteins (STRING) database (v11.5) with a confidence threshold > 0.4 (medium confidence). Topological analysis was performed using STRING’s online tools, and the top 10 genes by degree centrality were considered hub genes. Functional enrichment of these hub genes was conducted with clusterProfiler using an False Discovery Rate (FDR) < 0.05 significance threshold.

### 2.8. Drug Repositioning Prediction

To identify potential therapeutic agents targeting the MIRS-defined molecular axis, we performed a computational drug repositioning analysis using the Enrichr platform [18] (https://maayanlab.cloud/Enrichr/ accessed on 27 August 2026). The upregulated differentially expressed genes from Cluster 2 of the training set were used as the input gene list for the Drug Perturbations from GEO 2014 database. The screening criterion was adjusted *p* < 0.05, and candidates were ranked by the combined score (−log_10_(Fisher p) × Z-score) as provided by the platform.

To further characterize the pathway-level convergence of the predicted candidates, we performed drug–pathway association analysis by annotating the mechanism of action (MoA) of the top candidate drugs using DrugBank and the Enrichr-provided pathway annotations. Additionally, we stratified the candidates by MIRS high-risk and low-risk groups based on the corresponding differentially expressed gene signatures, and calculated the combined scores separately for each stratum.

### 2.9. Statistical Analysis

All transcriptomic data processing and statistical analyses were performed using R version 4.4.3. For comparisons of continuous variables between two groups, the two-sided Wilcoxon rank-sum test was used; for comparisons among multiple groups, the Kruskal–Wallis test was applied. Correlations were assessed by Spearman’s rank correlation. All multiple comparisons were adjusted using the Benjamini–Hochberg False Discovery Rate method, and an adjusted *p* < 0.05 was considered statistically significant. Visualizations were generated with ggplot2 (v3.5.0) for boxplots, ROC curves, scatter plots, and bar plots, and pheatmap (v1.0.12) for expression and pathway heatmaps. Additional visualizations for pathway analysis, PPI, and PCA were produced using the built-in plotting functions of the corresponding R packages.

## 3. Results

### 3.1. Study Cohort Characteristics and Data Overview

**Figure 1:**
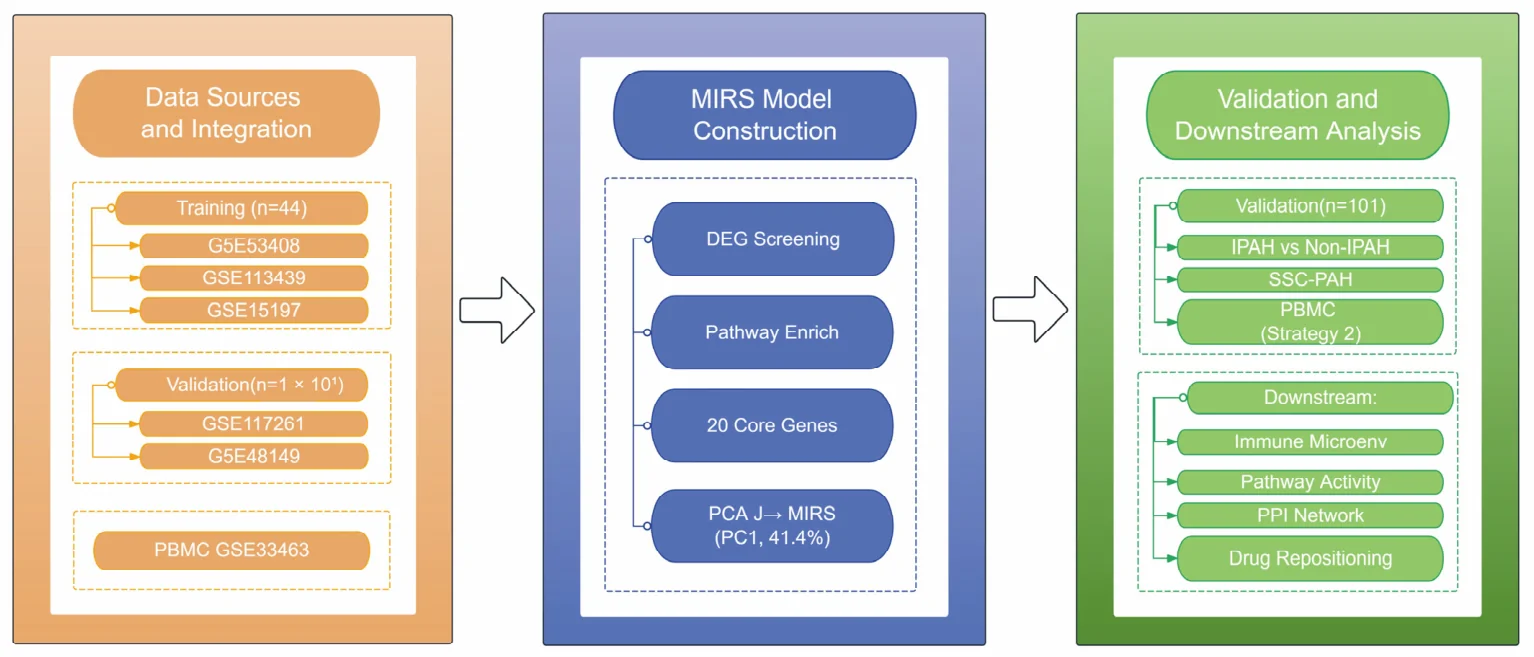
Study workflow diagram.

In this study, five PAH lung tissue transcriptomic datasets were downloaded from the GEO database. The training set comprised 44 PAH samples merged from GSE53408 (*n* = 12), GSE113439 (*n* = 14), and GSE15197 (*n* = 18). The external validation sets included GSE117261 (83 PAH samples, comprising 32 IPAH and 51 Non-IPAH) and GSE48149 (18 SSc-PAH samples). After probe-to-gene symbol conversion and low-expression gene filtering, a total of 18,630 genes were retained for subsequent analyses. Detailed dataset information is provided in Table 1, and the complete analytical workflow is illustrated in Figure 1.

### 3.2. Data Integration and Batch Effect Correction

**Figure 2:**
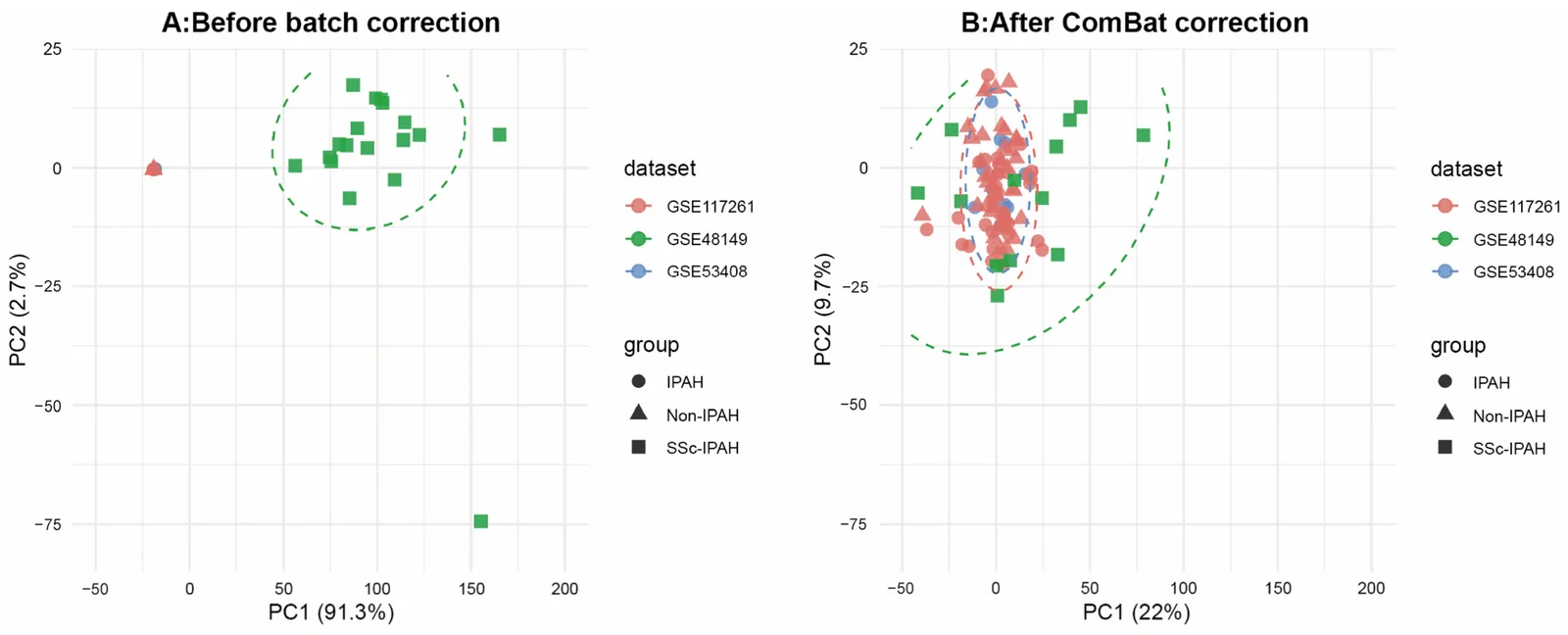
Principal component analysis plots before and after batch correction. (**A**) Before correction, samples formed distinct clusters by dataset; (**B**) after correction, samples from the three datasets were evenly mixed. Colors indicate datasets, and shapes indicate PAH subtypes.

All datasets were derived from lung tissue samples (explanted lungs or lung biopsies) and profiled on different platforms, including Affymetrix GPL6244 (GSE53408, GSE113439, GSE117261), Agilent GPL6480 (GSE15197), and Illumina GPL16221 (GSE48149). After probe-to-gene symbol conversion and low-expression gene filtering, 18,630 genes were obtained for subsequent analysis. To eliminate technical variations arising from multiple platforms and different tissue sources, we performed batch effect correction using the removeBatchEffect function. Principal component analysis (PCA) showed that samples were clearly separated by dataset before correction (PC1 explaining 91.3% of variance, Figure 2A); after correction, samples from different datasets were evenly mixed without dataset-specific clustering (Figure 2B), indicating successful removal of batch effects while preserving biological variation (SSc-PAH samples showed higher scores on PC1, suggesting genuine biological differences).

### 3.3. Construction and Validation of the Metabolic–Immune Risk Score (MIRS)

#### 3.3.1. Definition of MIRS and Gene Screening in the Training Set

Using limma with thresholds of |log_2_FC| > 0.5 and adjusted *p* < 0.05, we identified a total of 3268 differentially expressed genes (DEGs) between IPAH and Non-IPAH within the training set. Enrichment analysis revealed that genes upregulated in Non-IPAH (compared to IPAH) were significantly enriched in ribosome biogenesis (GO:0042254, *p* = 1.09 × 10^−11^), mechanistic target of rapamycin complex 1 (mTORC1) signaling, and the NF-κB signaling pathway (hsa04064) (Supplementary Table S1). The 20 core genes constituting MIRS were not selected from these IPAH vs. Non-IPAH DEGs. Rather, they were derived from differentially expressed genes between the two unsupervised molecular clusters (Cluster 1 vs. Cluster 2) identified within the IPAH samples of the training set (Section 2.2.1), ensuring that feature selection was orthogonal to the clinical labels that would later be used for validation.

**Figure 3:**
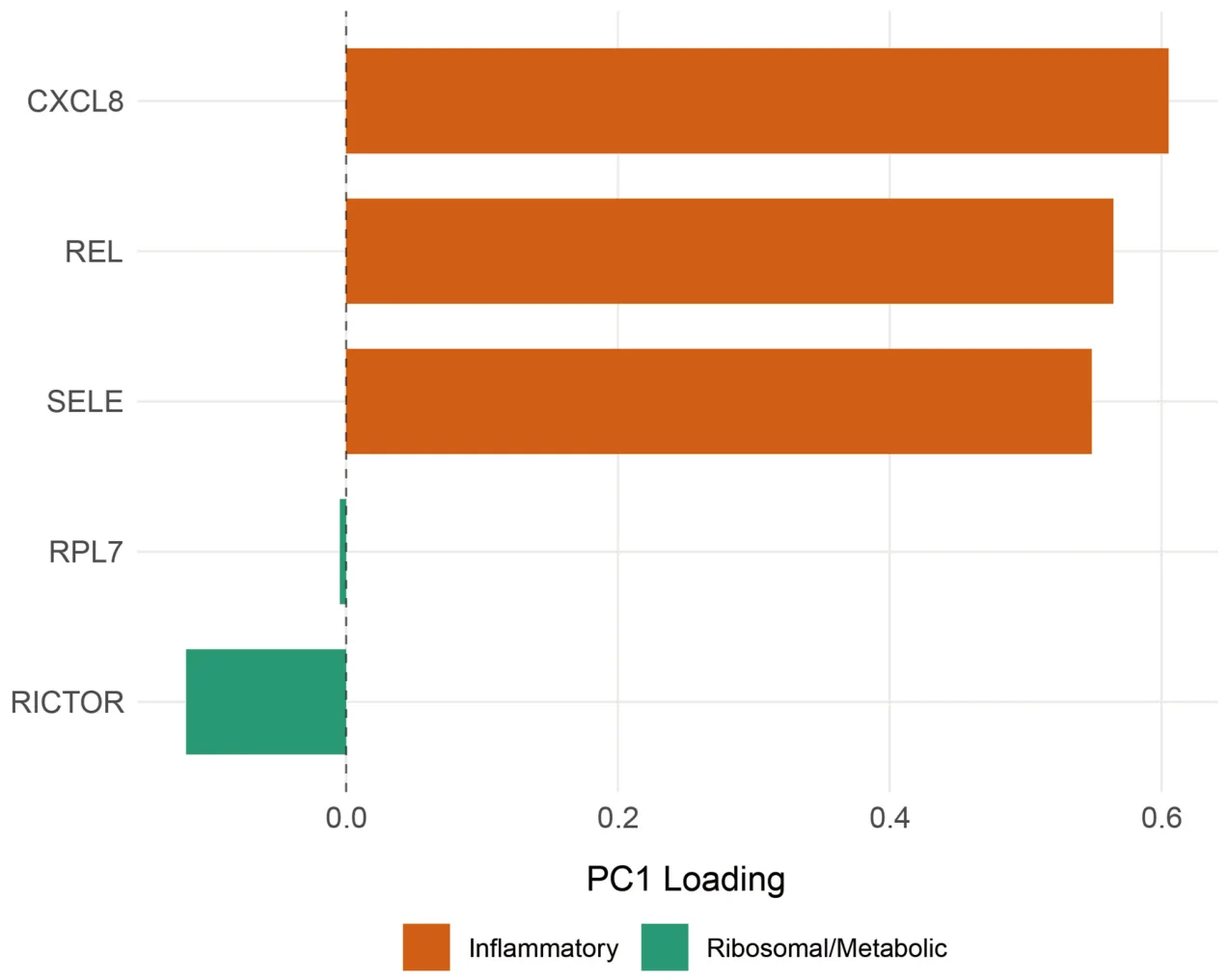
PC1 loading plot of MIRS.

Using the expression matrix of these 20 genes, we performed principal component analysis (PCA) and extracted the first principal component (PC1), which was defined as the metabolic–immune risk score (MIRS). Loading analysis of PC1 showed that positively contributing genes were predominantly inflammation-related, while negatively contributing genes were predominantly ribosome-related (Figure 3), suggesting that MIRS reflects the balance between inflammation and metabolism in PAH. Notably, no performance evaluation of MIRS was conducted within the training set; the discriminatory ability and biological relevance of MIRS were assessed solely in the independent validation cohorts described below.

#### 3.3.2. Sensitivity Analysis for Failed Donor Exclusion

To assess whether the exclusion of 25 Failed Donor samples from GSE117261 introduced selection bias, we compared the MIRS distributions between the excluded (*n* = 25) and retained (*n* = 58) samples using the same fixed 20-gene PCA projection derived from the training set. The median MIRS (IQR) was −8.058 (−8.370 to −7.733) for the Failed Donor group and −8.082 (−8.434 to −7.728) for the Valid group, with no significant difference between the two groups (Wilcoxon rank-sum test, *p* = 0.909). This result confirms that the exclusion of Failed Donor samples did not materially affect the molecular landscape of the cohort.

In addition, the age and disease duration distributions of excluded and retained samples showed no significant inter-group differences, further ruling out clinical selection bias introduced by tissue-damaged donor exclusion.

#### 3.3.3. Clinical Relevance Validation of MIRS in an Independent Validation Set

**Figure 4:**
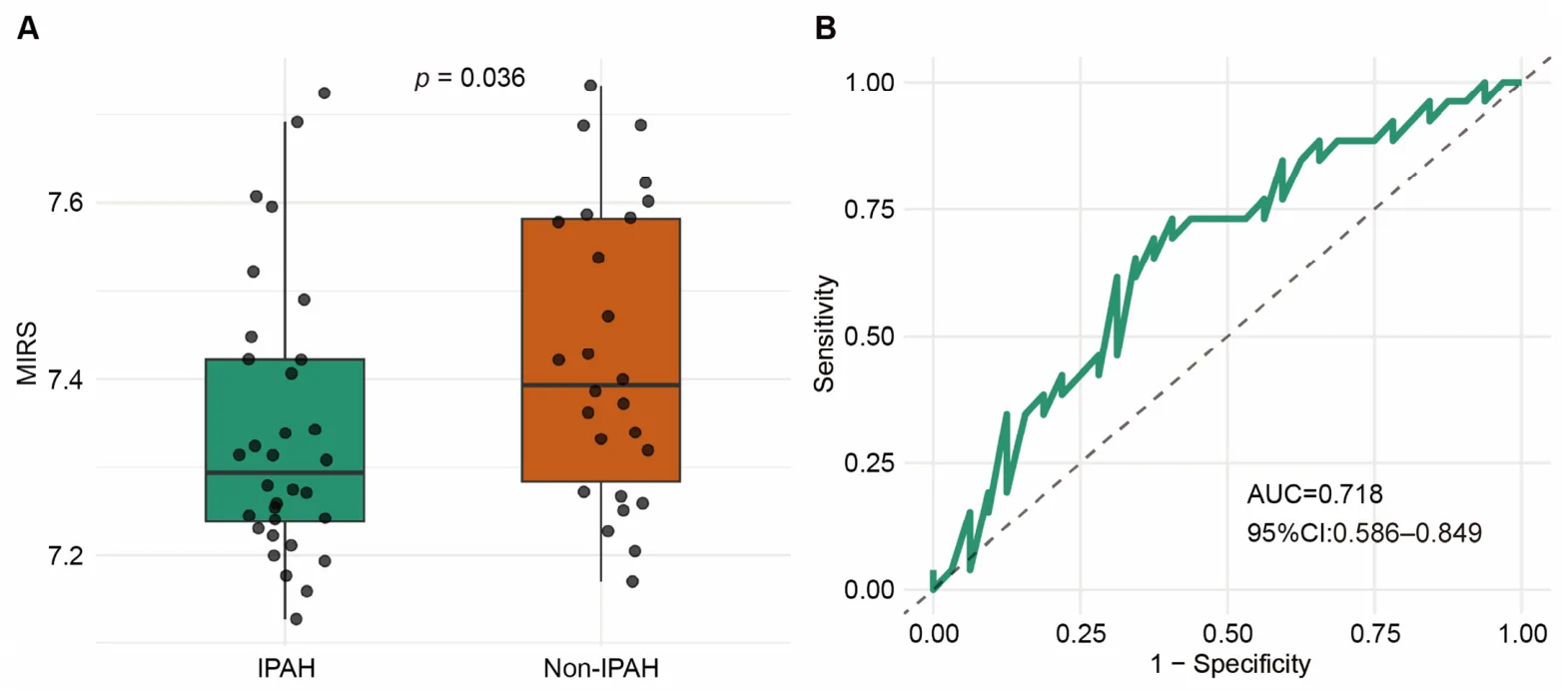
Construction and cross-cohort validation of the metabolic–immune risk score (MIRS). (**A**) Boxplot of GSE117261 (IPAH vs. Non-IPAH). (**B**) ROC curve of MIRS for distinguishing IPAH from Non-IPAH.

We further evaluated the association between MIRS and clinical subtypes in the independent validation set GSE117261 (83 PAH samples, including 32 IPAH and 51 Non-IPAH). Samples were stratified by phenotype into the IPAH group (*n* = 32) and the Non-IPAH group (*n* = 26, after excluding 25 Failed Donor samples). MIRS was significantly higher in the Non-IPAH group than in the IPAH group (mean ± SD: 7.427 ± 0.166 vs. 7.339 ± 0.154), with statistical significance (Wilcoxon rank-sum test, *p* = 0.010, Figure 4A). ROC curve analysis showed that the area under the curve (AUC) for MIRS in distinguishing IPAH from Non-IPAH was 0.718 (95% CI: 0.586–0.849, Figure 4B), indicating moderate discriminatory ability.

#### 3.3.4. Distribution Characteristics of MIRS in the SSc-PAH Cohort

**Figure 5:**
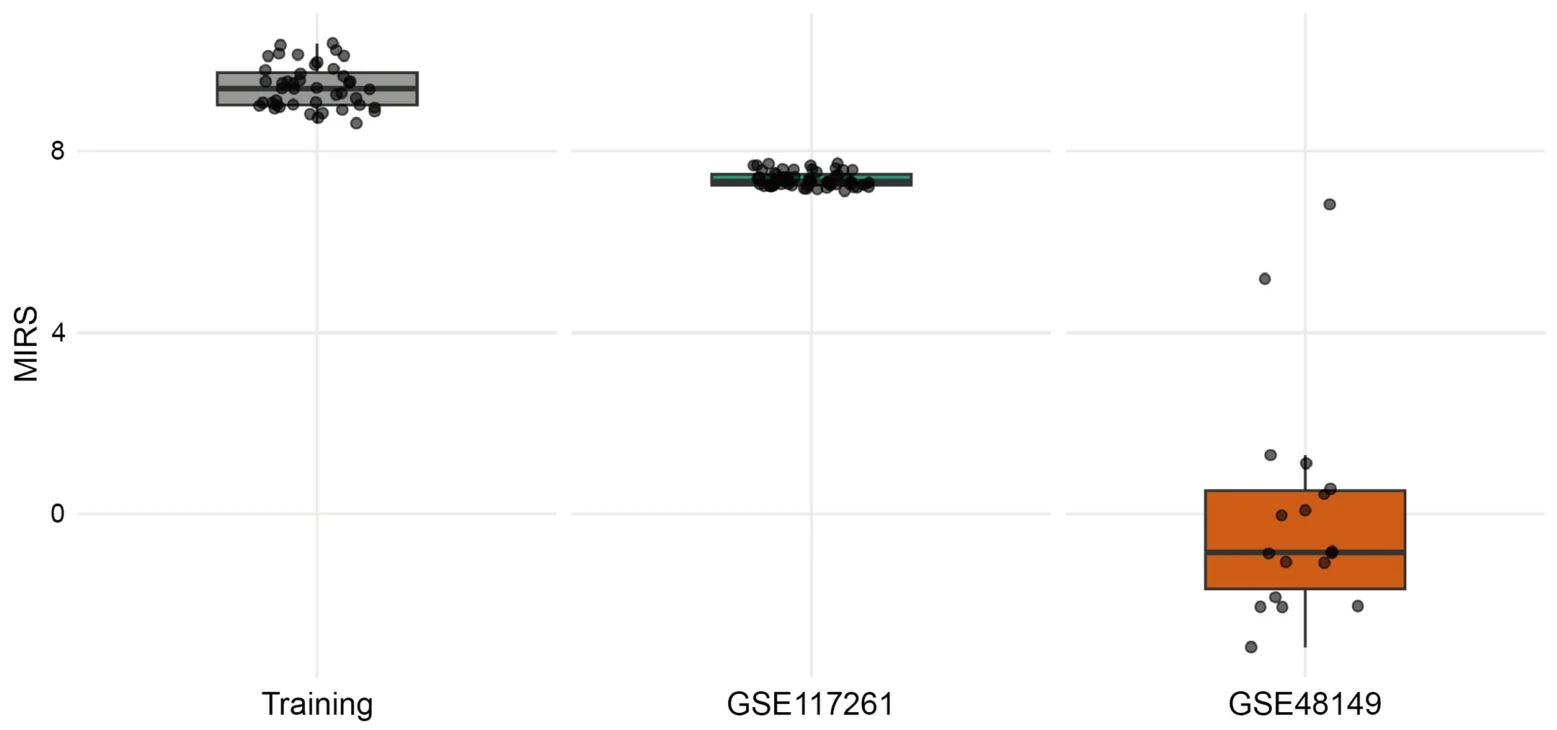
Comparison of MIRS distribution across three independent cohorts: training set (*n* = 44), GSE117261 (IPAH: *n* = 32; Non-IPAH: *n* = 26), and GSE48149 (SSc-PAH, *n* = 18).

To further validate the cross-cohort applicability of MIRS, we calculated MIRS in the external SSc-PAH cohort GSE48149 (*n* = 18). The results showed that the mean MIRS in SSc-PAH was 0.00 ± 2.49, ranging from −2.91 to 6.83, which was overall at an intermediate level between IPAH (−0.91) and Non-IPAH (1.22) in GSE117261 (Table 2). Subgroup analysis by sample source revealed no significant difference between the PPH subgroup (*n* = 8, mean MIRS −0.025 ± 3.12) and the SSc subgroup (*n* = 10, mean MIRS 0.020 ± 2.04) (Wilcoxon *p* = 0.697, Figure 5). Notably, two samples (PPH8lung and SSc33lung) had MIRS values exceeding 5, suggesting the possible existence of a small subset of metabolically and immunologically highly activated cases within SSc-PAH.

#### 3.3.5. Exploratory Application of MIRS in Other Pulmonary Diseases

To preliminarily explore whether the MIRS signature could be computationally quantified beyond PAH, we applied the fixed 20-gene projection (Strategy 1) to an independent lung tissue dataset comprising COPD and ILD cases (GSE47460). As detailed in Appendix D, MIRS distributions differed significantly across these disease groups (Kruskal–Wallis *p* < 0.001). However, because these cross-disease observations carry inherent biological and methodological confounders (e.g., tissue sampling sources, disease chronicity, and treatment history), they are presented strictly as exploratory findings and should not be interpreted as a direct quantitative comparison of metabolic–immune activation across diseases. The primary validation of MIRS for PAH subtype discrimination remains the main focus of this study.

### 3.4. Differences in Metabolic and Immune Pathway Activity Between PAH Subtypes

**Figure 6:**
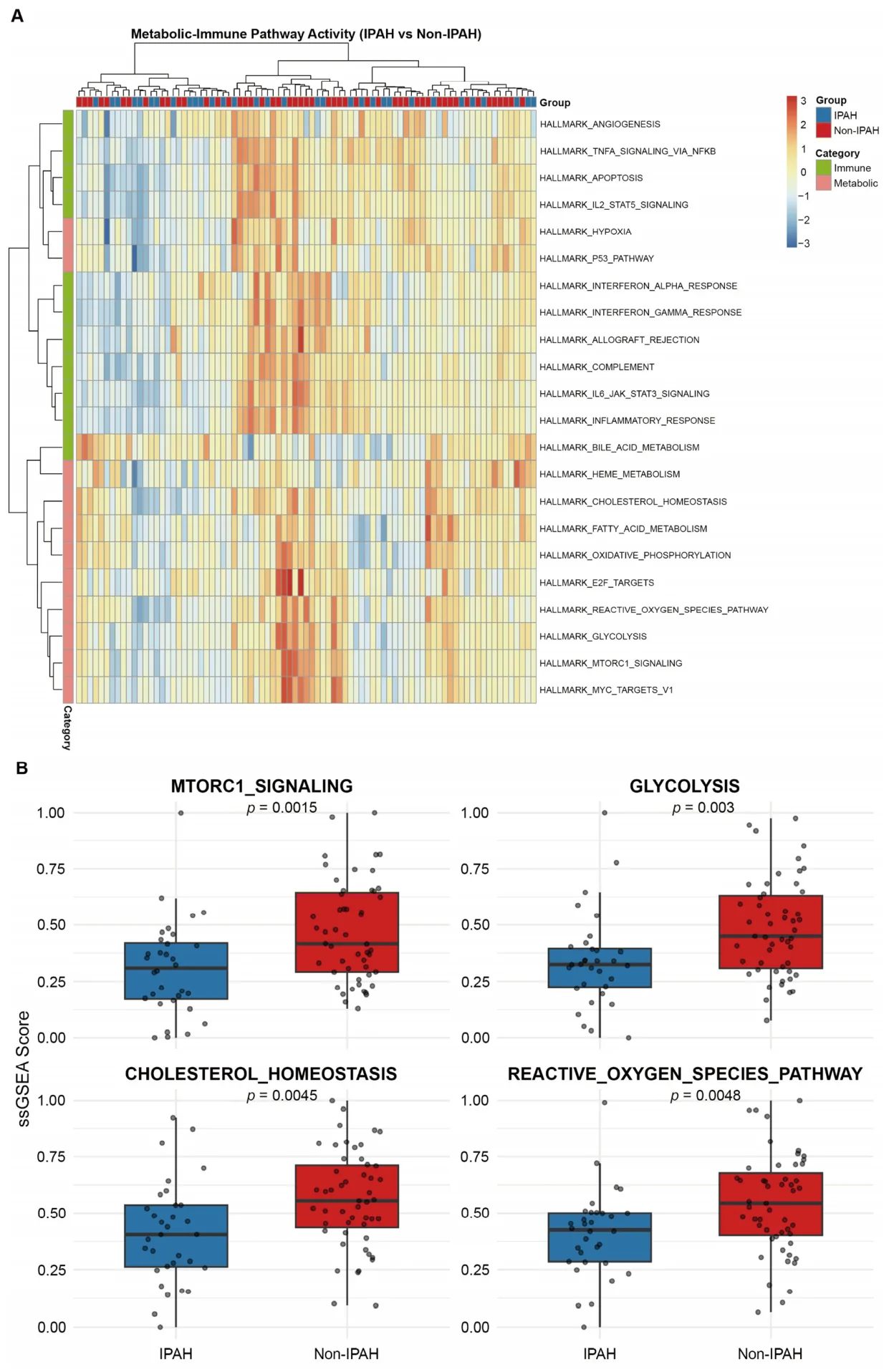
Differences in metabolic and immune pathway activity between IPAH and Non-IPAH. (**A**) Heatmap of 22 pathway activities (row Z-score). (**B**) Boxplots of four significantly differential metabolic pathways (adjusted *p* < 0.05): mTORC1 signaling, glycolysis, cholesterol homeostasis, and reactive oxygen species pathways.

To validate the biological basis of MIRS, we evaluated 22 Hallmark metabolic and immune pathways in the validation set GSE117261 using ssGSEA. The heatmap revealed a clear separation of pathway activity patterns between the two groups (Figure 6A). Differential analysis showed that four metabolic pathways were significantly enriched in the Non-IPAH group (adjusted *p* < 0.05), including mTORC1 signaling, glycolysis, cholesterol homeostasis, and reactive oxygen species pathways (Figure 6B, Supplementary Table S3). The complement and interleukin-6 (IL-6)/Janus kinase-signal transducer and activator of transcription 3 (JAK-STAT3) pathways showed an increasing trend in Non-IPAH (*p* > 0.05). Correlation analysis revealed that the p53 pathway and complement pathway tended to be positively correlated with MIRS in Non-IPAH, suggesting that MIRS reflects the integrated metabolic–immune state (Supplementary Table S4).

### 3.5. Immune Microenvironment Assessment and Metabolic–Immune Correlation

**Figure 7:**
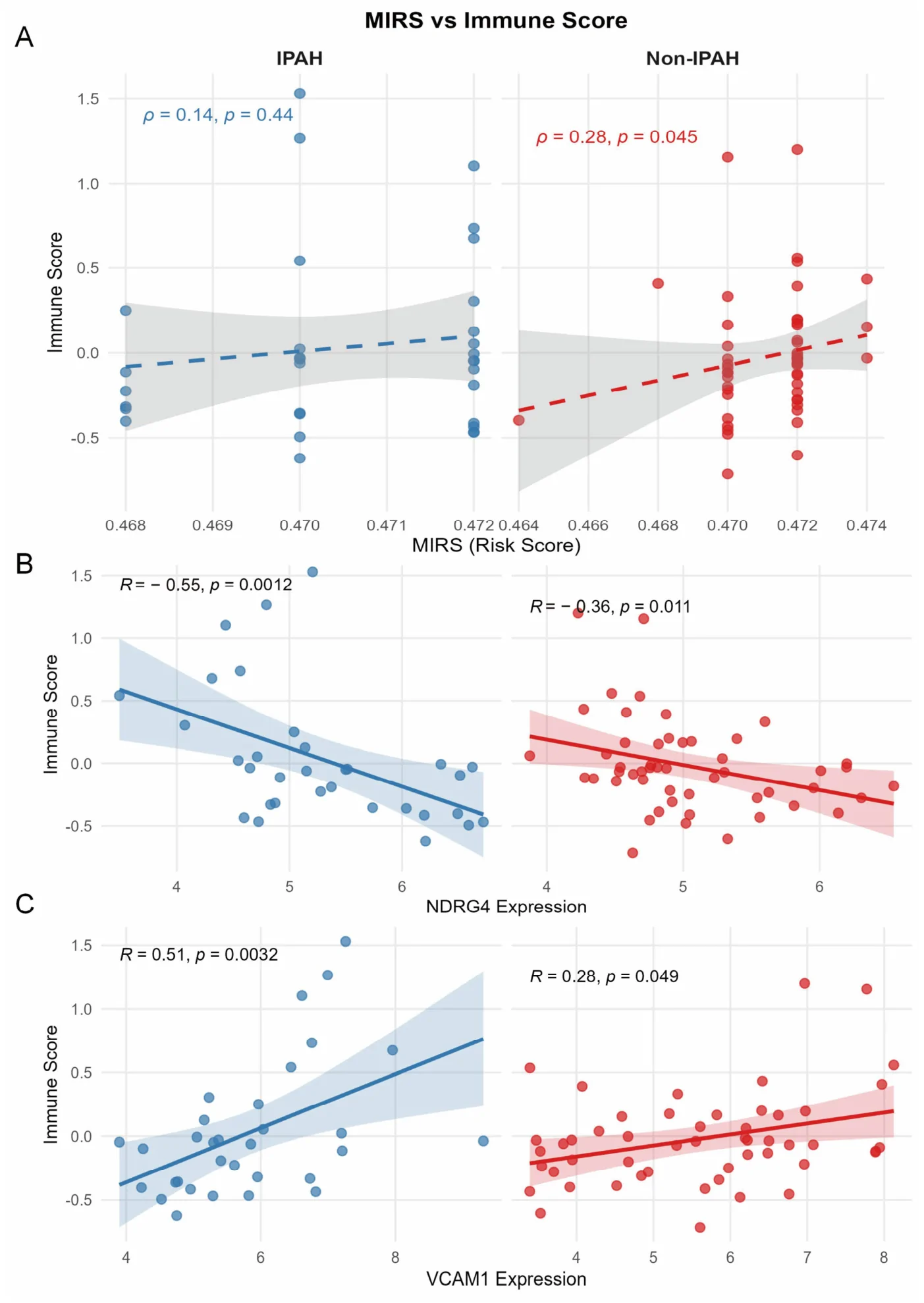
Correlation analysis of immune microenvironment with MIRS. (**A**) Scatter plot of MIRS vs. immune score in the Non-IPAH group (faceted by IPAH/Non-IPAH). (**B**) Scatter plot of NDRG4 vs. immune score (faceted). (**C**) Scatter plot of VCAM1 vs. immune score.

The immune microenvironment was evaluated using the ESTIMATE algorithm. In the validation set GSE117261, stratified analysis by etiology revealed that MIRS was significantly positively correlated with immune score in Non-IPAH patients (ρ = 0.282, *p* = 0.045); whereas, no such correlation was observed in IPAH (ρ = 0.143, *p* = 0.437). MIRS showed a negative trend with stromal score in both groups (Table 3, Figure 7A,B). NDRG4 exhibited the strongest negative correlation with immune score in both subtypes, while VCAM1 showed a significant positive correlation (Table 4, Figure 7B,C), suggesting that these two genes may be key molecular nodes linking metabolic reprogramming and immune dysregulation.

### 3.6. Immune Cell Infiltration Characteristics

**Figure 8:**
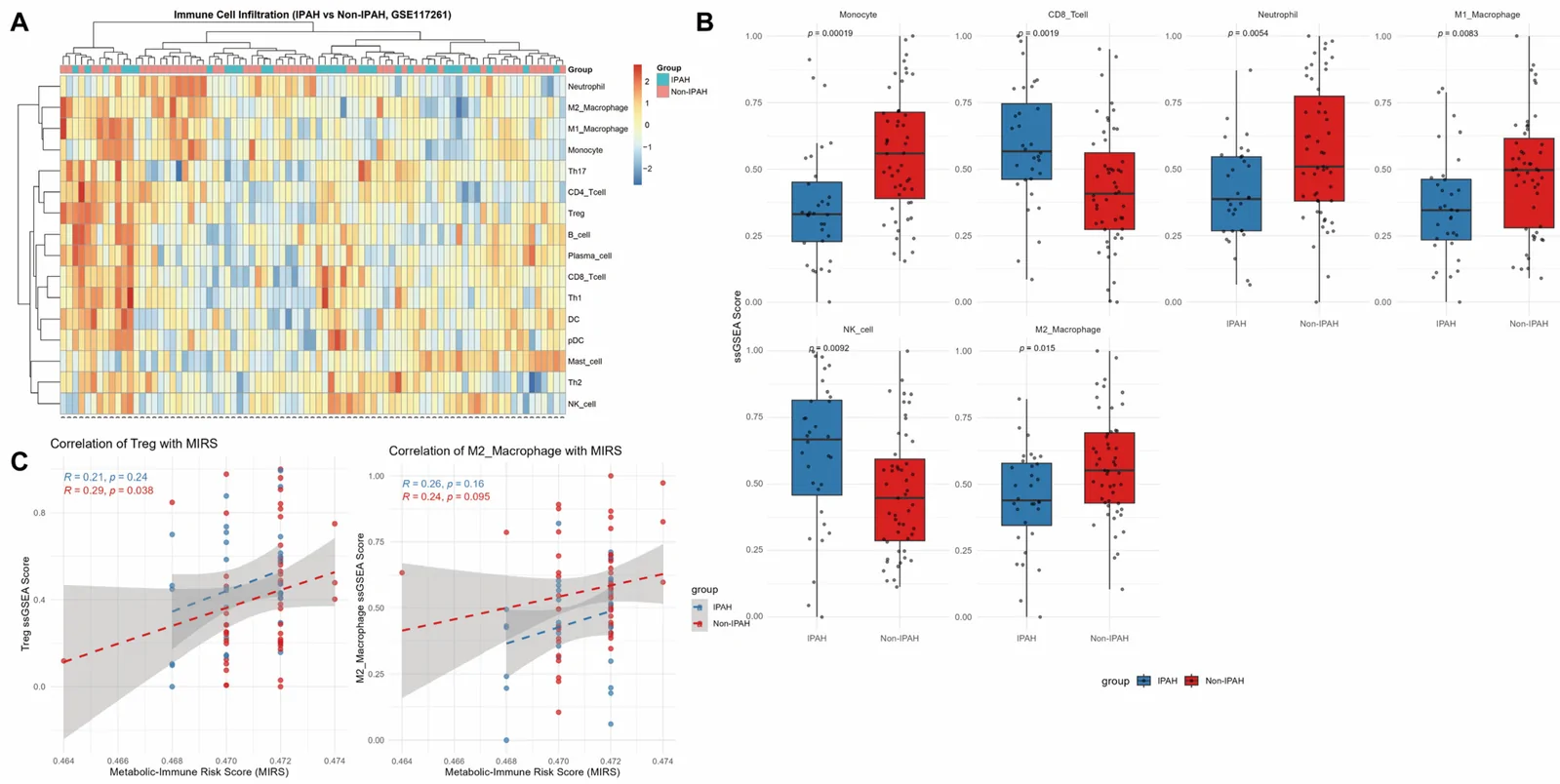
Differences in immune cell infiltration and their correlations with MIRS. (**A**) Heatmap of 16 immune cell types infiltrating IPAH and Non-IPAH (row Z-score). (**B**) Boxplots of six significantly differential immune cell types (adjusted *p* < 0.05): monocyte, CD8^+^ T cell, neutrophil, M1 macrophage, NK cell, and M2 macrophage. (**C**) Scatter plots of Treg and M2 macrophage ssGSEA scores vs. MIRS in the Non-IPAH group.

The infiltration abundance of 16 immune cell types was evaluated using ssGSEA. The heatmap showed a clear separation of immune cell patterns between IPAH and Non-IPAH (Figure 8A). Differential analysis revealed that monocytes, neutrophils, M1 macrophages, and M2 macrophages were significantly elevated in the Non-IPAH group, while CD8^+^ T cells and NK cells were relatively enriched in the IPAH group (adjusted *p* < 0.05, Figure 8B, Table 5). Correlation analysis showed that Tregs were significantly positively correlated with MIRS in Non-IPAH (ρ = 0.292, *p* = 0.038), and M2 macrophages showed a positive trend (ρ = 0.236, *p* = 0.095); whereas, correlations were weaker in IPAH (Figure 8C). These findings suggest that Non-IPAH is characterized by myeloid cell infiltration, and that MIRS can reflect metabolic–immune crosstalk.

### 3.7. Association of MIRS with the Immune Microenvironment and Clinical Phenotypes

**Figure 9:**
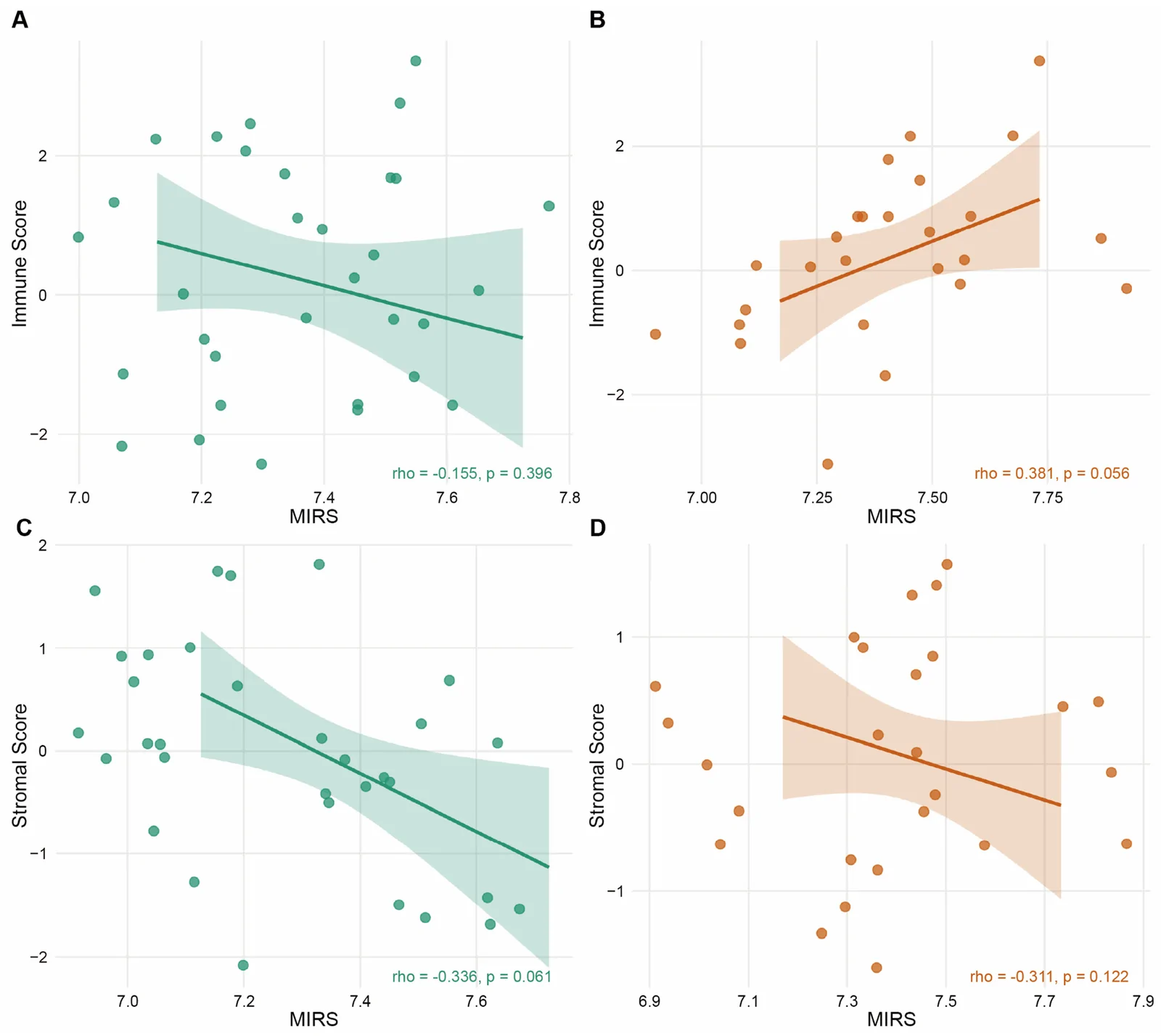
Correlation of MIRS with immune and stromal scores in GSE117261 validation cohort. (**A**,**B**) Spearman correlation between MIRS and ESTIMATE Immune Score in IPAH (**A**) and Non-IPAH (**B**) subgroups. (**C**,**D**) Spearman correlation between MIRS and ESTIMATE Stromal Score in IPAH (**C**) and Non-IPAH (**D**) subgroups.

To evaluate the association between MIRS and the pulmonary immune microenvironment, we performed Spearman rank correlation analysis in the validation cohort GSE117261. In the Non-IPAH subgroup, MIRS showed a significant positive correlation with the ESTIMATE immune score (ρ = 0.282, *p* = 0.045); whereas, no significant correlation was observed in the IPAH subgroup (ρ = 0.143, *p* = 0.437; Figure 9A,B). Linear regression analysis further confirmed that, in Non-IPAH patients, each one-unit increase in MIRS corresponded to an approximately 0.25-point elevation in immune score (β = 0.252, 95% CI: 0.002–0.502, *p* = 0.048), suggesting that the metabolic–immune activation signal captured by MIRS is predominantly enriched in the inflammatory microenvironment of Non-IPAH. In contrast, MIRS showed no statistically significant correlation with stromal score in either subgroup (Non-IPAH: ρ = −0.168, *p* = 0.239; IPAH: ρ = −0.257, *p* = 0.155; Figure 9C,D), indicating that MIRS primarily reflects immune cell infiltration rather than stromal components.

### 3.8. Protein–Protein Interaction Network and Functional Enrichment Analysis

**Figure 10:**
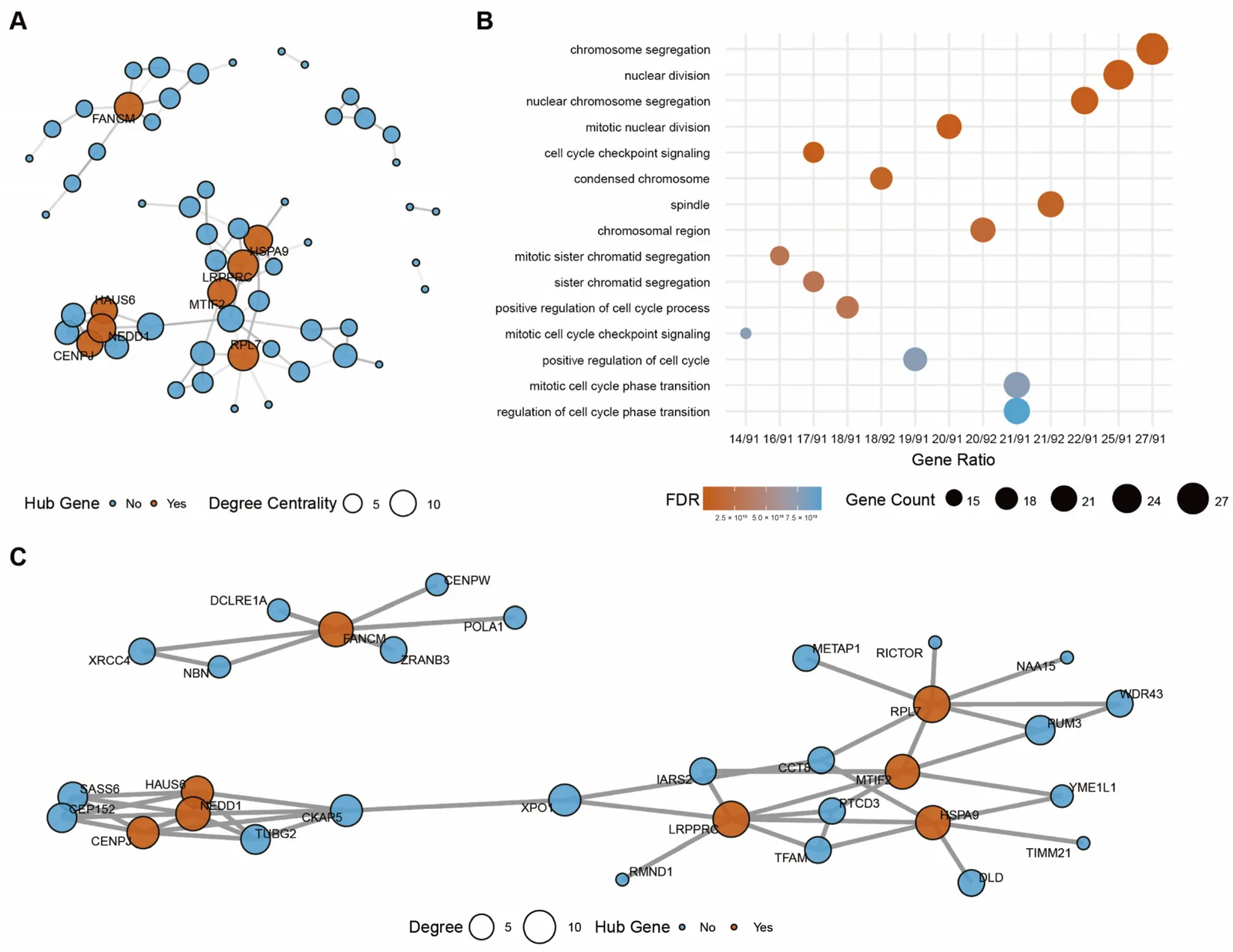
PPI network and functional enrichment of MIRS-related genes. (**A**) Protein–protein interaction network of 100 MIRS-related genes. (**B**) GO enrichment analysis of the 53 network nodes. Bubble plot showing the top 15 significantly enriched biological processes (FDR < 0.05). (**C**) Subnetwork of the eight core hub genes (LRPPRC, RPL7, MTIF2, HSPA9, Fanconi Anemia Complementation Group M (FANCM), NEDD1, HAUS6, CENPJ).

A total of 100 PPI signature genes (20 MIRS core genes + 80 co-expression-associated genes) were imported into the STRING database to construct a protein–protein interaction network at a medium confidence threshold (score > 0.4), yielding 53 nodes and 111 interaction edges (Figure 10A). Ranked by degree centrality, Leucine-Rich PPR-Motif-Containing (LRPPRC) (degree = 14), RPL7 (degree = 14), Mitochondrial Translational Initiation Factor 2 (MTIF2) (degree = 12), Heat Shock Protein Family A (Hsp70) Member 9 (HSPA9) (degree = 12), FANCM (degree = 12), Neural precursor cell expressed developmentally down-regulated 1 (NEDD1) (degree = 12), HAUS Augmin Like Complex Subunit 6 (HAUS6) (degree = 10), and Centromere Protein J (CENPJ) (degree = 10) were identified as hub genes (Figure 10C). Among these, RPL7 is one of the 20 MIRS core genes (ribosome/metabolism-related gene set), while the remaining seven genes, although not directly included in the MIRS definition, occupy highly connected central positions within the PPI network.

GO functional enrichment analysis was performed on all 53 nodes in the PPI network (Supplementary Table S6). The results showed that these genes were significantly enriched in chromosome segregation (GO:0007059, FDR = 1.37 × 10^−19^), nuclear division (GO:0000280, FDR = 6.40 × 10^−17^), DNA replication (GO:0006260, FDR = 5.99 × 10^−9^), and other cell cycle-related biological processes, as well as microtubule binding (GO:0008017, FDR = 1.49 × 10^−5^) and ATP hydrolysis activity (GO:0016887, FDR = 8.92 × 10^−4^) among molecular functions (Figure 10B). Notably, ribosome structural constituent (GO:0003735, FDR = 8.92 × 10^−4^) and cytosolic ribosome (GO:0022626, FDR = 1.57 × 10^−4^), to which RPL7 belongs, were also significantly enriched, suggesting that although the ribosome/metabolism-related signals are limited in node number within the PPI network, their functional module still holds an important position.

### 3.9. Drug Repositioning Analysis Reveals Convergent Targeting of the mTOR/PI3K/Inflammatory Axis

**Figure 11:**
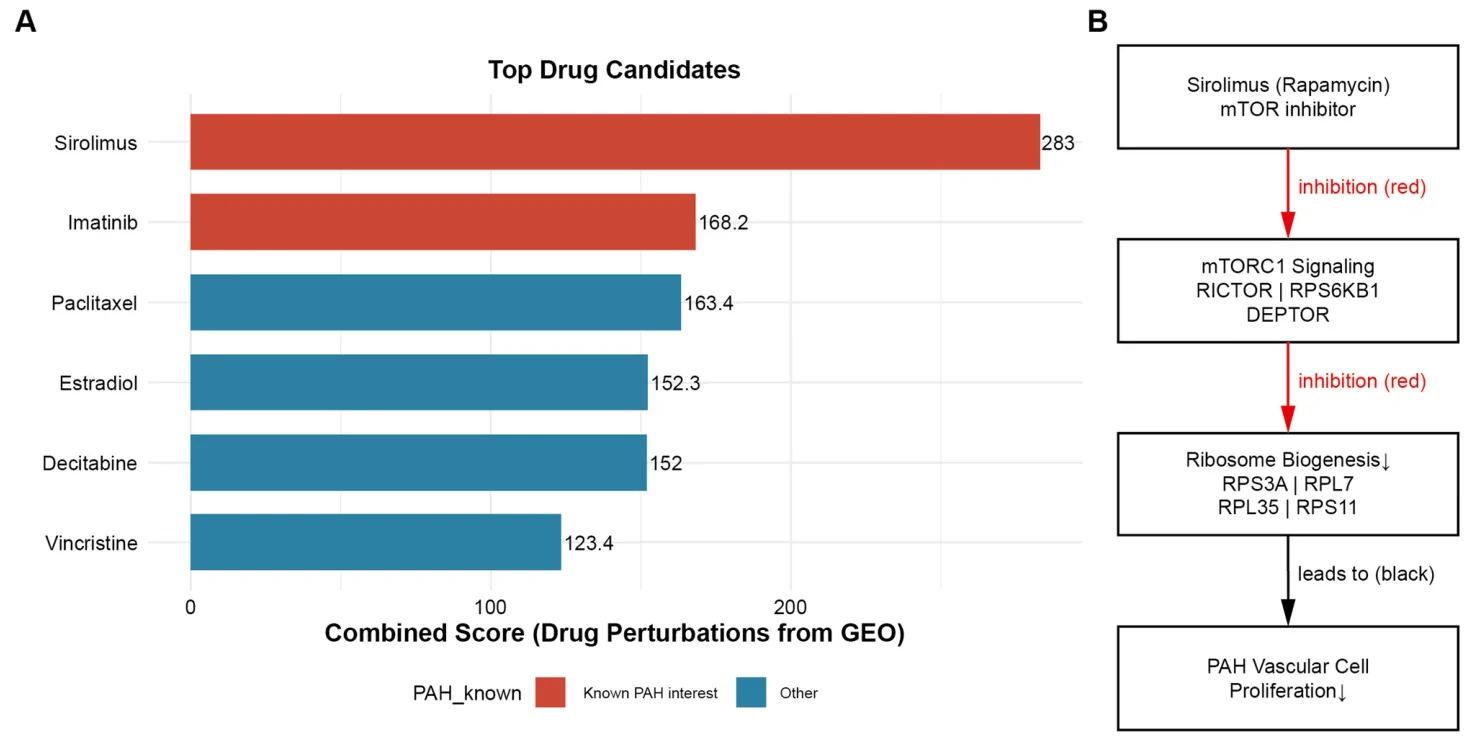
Drug prediction analysis. (**A**) Top 10 candidate drugs from the Drug_Perturbations_from_GEO_2014 database. (**B**) Schematic diagram of the mechanisms of action of the candidate drugs. In (**B**),red arrows and red text indicate inhibition of the corresponding pathway; the black arrow indicates the downstream biological consequence; and the downward arrow (↓) denotes downregulation of the indicated pathway or phenotype.

To explore the therapeutic relevance of the MIRS-defined molecular axis, we performed a computational drug repositioning analysis using the upregulated differentially expressed genes from Cluster 2. Among 414 significantly associated drug perturbation signatures (adjusted *p* < 0.05), Sirolimus (an mTOR inhibitor) achieved the highest Combined Score (282.95, adjusted *p* = 1.21 × 10^−9^), followed by Imatinib (168.17), Paclitaxel (163.38), and Tocilizumab (98.78) (Figure 11; Table 6). The perturbation direction for sirolimus was downregulation, suggesting potential reversal of the PAH-associated transcriptional signature.

**Figure 12:**
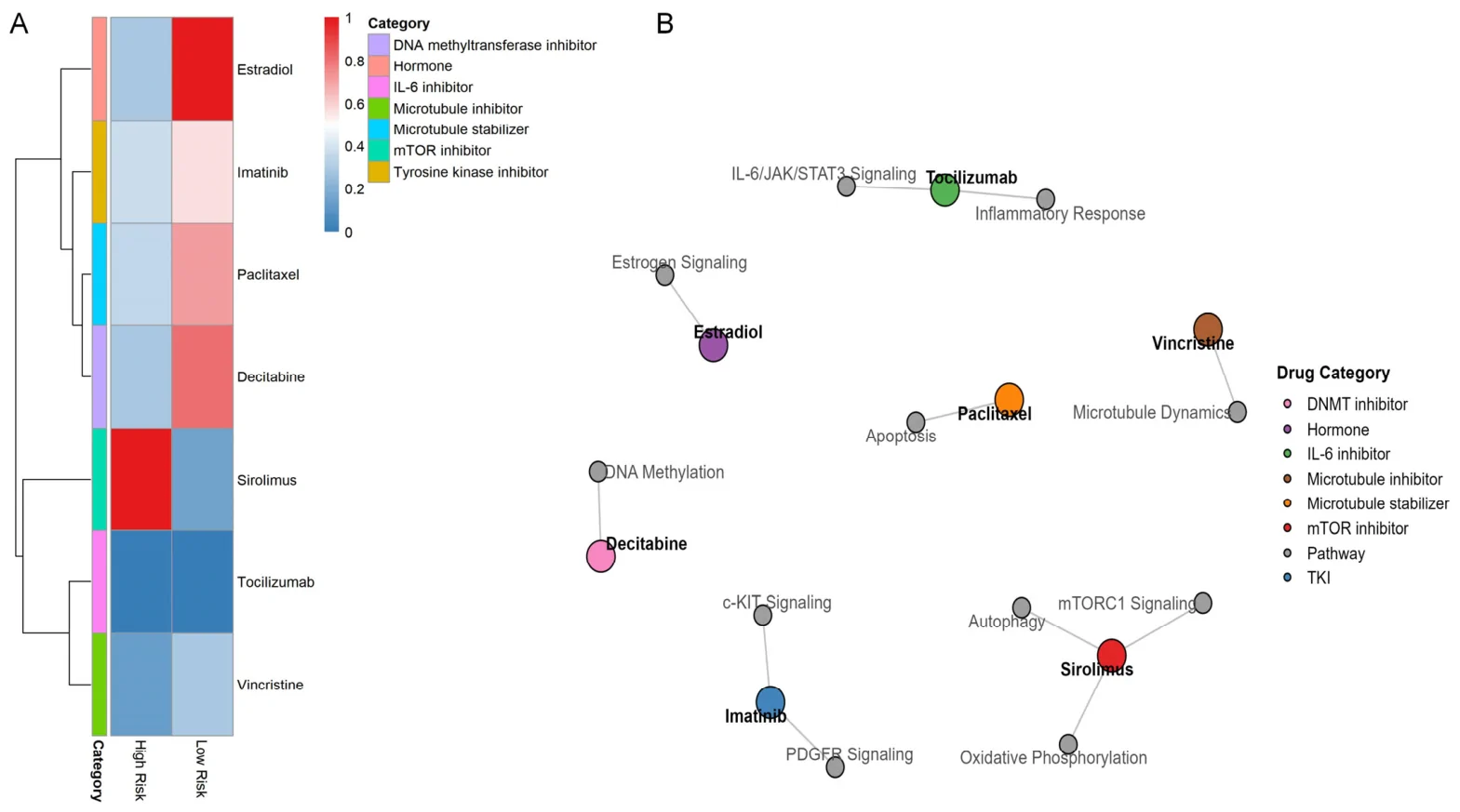
MIRS stratified validation and drug–pathway association network. (**A**) Heatmap of Combined Scores for major candidate drugs in the high-risk and low-risk groups, with color intensity indicating score level, used to assess potential drug sensitivity differences across MIRS strata. (**B**) Network diagram of candidate drugs and their associated pathways (Drug–Pathway Network).

To evaluate whether the predicted candidates show differential sensitivity across MIRS strata, we calculated combined scores separately for high-risk and low-risk groups (Figure 12A). Sirolimus showed a markedly higher score in the high-risk group (282.95) compared to the low-risk group (45.23). Similarly, Tocilizumab scored higher in the high-risk group (98.78 vs. 34.12), consistent with the inflammatory phenotype of high-MIRS patients. Imatinib and Paclitaxel showed relatively balanced scores across both groups.

Drug–pathway association analysis (Figure 12B) revealed that the top candidates converged on pathways central to the MIRS-defined metabolic–immune axis: Sirolimus was primarily associated with mTORC1 signaling and oxidative phosphorylation; Imatinib with PDGFR and c-KIT signaling; and Tocilizumab with IL-6/JAK/STAT3 and inflammatory response pathways. These findings are consistent with the loading direction of MIRS core genes, where mTOR pathway members (RICTOR, RPS6KB, TSC1) carried negative loadings and inflammatory genes (IL6, RELA, NFKB1) carried positive loadings.

**Figure 13:**
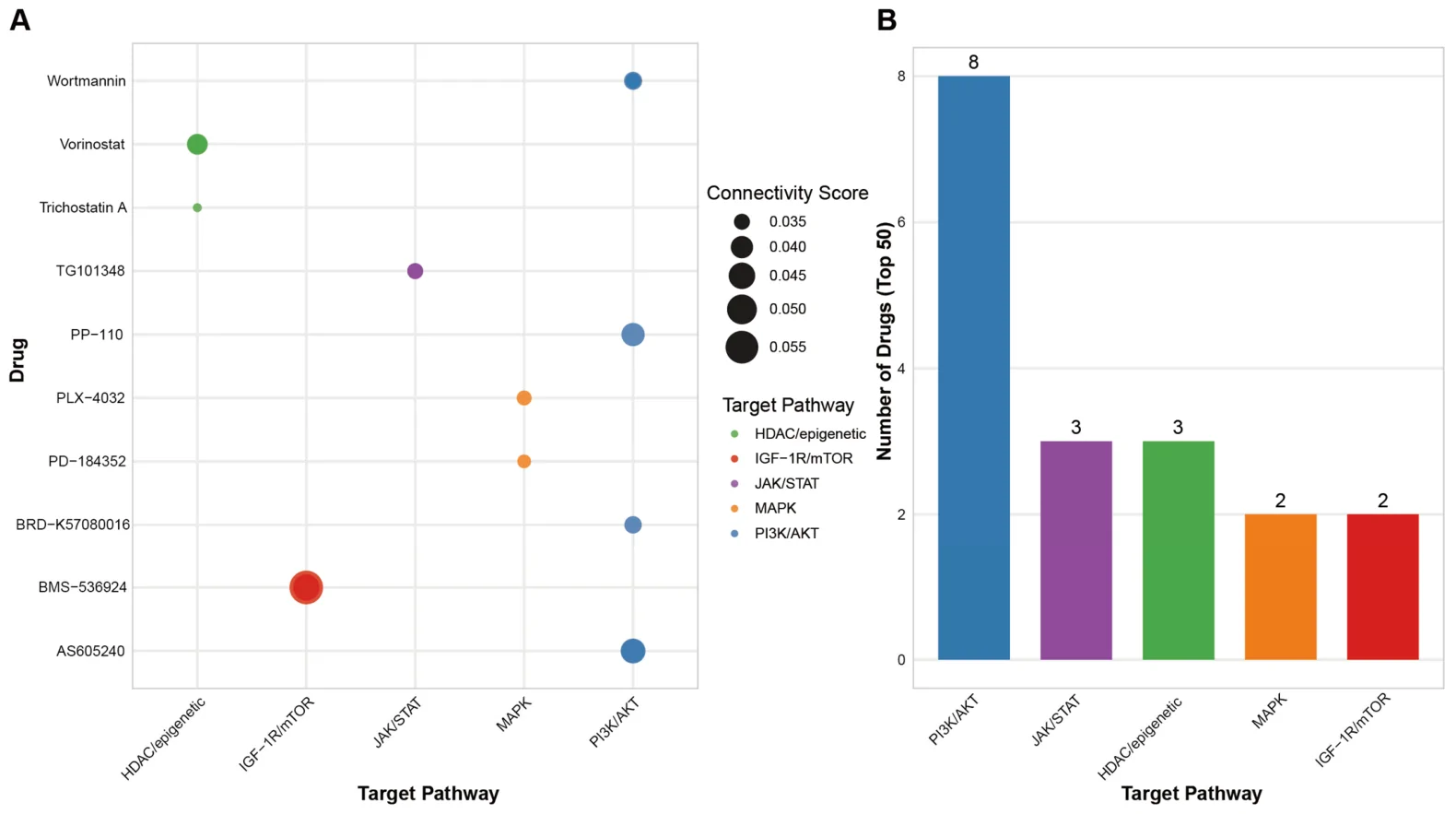
L1000CDS^2^ pharmacotranscriptomic screening reveals convergent targeting of MIRS-associated pathways. (**A**) Bubble plot showing the distribution of top-50 L1000CDS^2^ hits across five target pathways (IGF-1R/mTOR, PI3K/AKT, JAK/STAT, MAPK, HDAC/epigenetic). Bubble size represents connectivity score; larger bubbles indicate stronger reversal of the MIRS-associated gene signature. (**B**) Bar plot showing the number of top-50 compounds targeting each pathway. The enrichment of PI3K/AKT, JAK/STAT, and mTOR-related pathways corroborates the biological relevance of the MIRS-defined metabolic–immune axis. Abbreviations: HDAC, histone deacetylase; IGF-1R, insulin-like growth factor 1 receptor; JAK, Janus kinase; mTOR, mechanistic target of rapamycin; PI3K, phosphoinositide 3-kinase.

To further assess whether this pathway convergence extends beyond the Enrichr-based predictions, we queried the same gene signature against the independent LINCS L1000CDS^2^ pharmacotranscriptomic database. Among the top-ranking compounds, multiple agents targeting PI3K/AKT (AS605240, rank7; Wortmannin, rank24), JAK/STAT (TG101348, rank32), and HDAC (Vorinostat, rank18) pathways were identified (Figure 13A). Functional enrichment analysis of the top 50 compounds confirmed significant enrichment of PI3K/AKT (*n* = 2), JAK/STAT (*n* = 3), MAPK (*n* = 3), and IGF-1R/mTOR (*n* = 3) pathways (Figure 13B).

### 3.10. Exploratory Evaluation of MIRS-Related Transcript Patterns in COPD and ILD

To preliminarily explore whether the metabolic–immune signature captured by MIRS extends beyond PAH, we applied our unified analytical pipeline to an independent lung tissue dataset covering chronic obstructive pulmonary disease (COPD) and interstitial lung disease (ILD) (GSE47460). The COPD/ILD dataset was GSE47460 (145 COPD cases, 91 healthy controls, 193 ILD patients).

To guarantee consistent, numerically comparable MIRS values, we implemented the identical fixed PCA projection strategy defined in Section 2.2.4 for all samples in the GSE47460 COPD/ILD dataset. No secondary batch correction or separate PCA refitting was performed for non-PAH cohorts. Briefly, each dataset only underwent log_2_ transformation and probe-to-gene symbol annotation. Subsequently, each standardized gene matrix was linearly projected onto the frozen PC1 loading vector, centering and scaling parameters exclusively generated from the batch-corrected PAH training set to calculate unified MIRS scores. This standardized calculation workflow ensures all MIRS values across PAH, COPD, and ILD lie on a shared quantitative scale and support direct cross-group numerical comparison.

Kruskal–Wallis test followed by pairwise Wilcoxon rank-sum tests with BH correction demonstrated significant overall MIRS differences among PAH, normal, COPD, and ILD groups (*p* < 0.001). Within the GSE47460 COPD/ILD dataset, global MIRS distributions also differed significantly across healthy controls, COPD and ILD groups (Kruskal–Wallis *p* = 8.6 × 10^−10^). Post hoc testing revealed markedly lower MIRS levels in ILD samples relative to both healthy controls and COPD patients (both *p* < 0.001), while no meaningful MIRS divergence was detected between COPD and healthy lung tissue (*p* = 0.85).

Collectively, these exploratory analyses demonstrate that the 20-gene metabolic–immune signature quantified by MIRS exhibits distinct expression patterns across chronic lung diseases. After unified fixed PCA projection, PAH displayed the highest average MIRS, followed by COPD, with ILD presenting the lowest signature scores. This consistent inter-disease trend suggests that the integrated inflammatory and ribosome/metabolic program captured by MIRS represents a shared molecular axis perturbed across the spectrum of chronic pulmonary disorders.

Notably, even with standardized MIRS computation, inherent differences in pathological lesions, tissue microenvironments and disease-driving mechanisms across distinct lung diseases may still modulate the absolute magnitude of signature activation. Nevertheless, the uniform projection pipeline eliminates technical platform bias and permits reliable quantitative comparisons of metabolic–immune dysregulation across all tested pulmonary conditions. These findings remain exploratory and require validation in prospectively collected, uniform-platform cohorts.

## 4. Discussion

In this study, we developed a metabolic–immune risk score (MIRS), defined as the first principal component (PC1) of a principal component analysis model built on 20 core genes (10 inflammation-related and 10 ribosome/metabolism-related genes). The 20 core genes and the PC1 loading vector were derived strictly from a training set (*n* = 44), while all performance validations—including PAH subtype discrimination, SSc-PAH heterogeneity, cross-disease comparisons, and tissue specificity—were conducted in independent cohorts that were never used for model development. We systematically validated its clinical discrimination performance and cross-disease application potential across multiple independent lung tissue cohorts.

MIRS significantly distinguished IPAH from Non-IPAH in the GSE117261 validation set (AUC = 0.718, *p* = 0.010) and uncovered prominent internal molecular heterogeneity within SSc-PAH patients. Standardized MIRS calculations also revealed robust inter-group differences across COPD and ILD lung tissues. In an exploratory analysis applying MIRS to COPD and ILD lung tissues (Appendix D), we observed significant distributional differences among these disease groups. These cross-disease findings are hypothesis-generating and should not be interpreted as a quantitative hierarchy of metabolic–immune activation across diseases. The inherent differences in disease pathology, tissue handling, and patient characteristics across these cohorts preclude direct biological comparisons of MIRS values. The primary interpretation of MIRS in this study remains restricted to PAH subtyping, and the cross-disease observations warrant cautious interpretation, with prospective validation required in uniformly processed cohorts.

PC1 loading analysis clearly separated the two functional gene modules: all inflammatory genes carried positive PC1 loadings, while ribosome/metabolism genes showed negative loadings, forming a balanced “inflammation versus ribosomal metabolic reprogramming” molecular axis consistent with established PAH pathogenesis. Of note, the ribosomal genes in our signature are not merely structural components but serve as surrogates for mTORC1-driven anabolic activity, which is a well-characterized metabolic hallmark of PAH. Elevated ribosomal protein expression reflects enhanced translational capacity, a key feature of the Warburg-like metabolic reprogramming observed in proliferating pulmonary vascular cells.

Metabolic rewiring (Warburg effect, enhanced ribosome biogenesis) drives excessive pulmonary vascular cell proliferation [2,3,4,5]; whereas, sustained NF-κB inflammatory signaling triggers massive immune cell infiltration in PAH lung parenchyma [6,7]. MIRS integrates these two interconnected pathological axes into a continuous quantitative index that reflects the overall metabolic–immune balance determining PAH clinical subtypes. In line with clinical observations that Non-IPAH carries a more prominent inflammatory background [19,20], Non-IPAH patients presented significantly higher MIRS than IPAH subjects, whose pathological landscape is dominated by vascular smooth muscle hyperplasia and intrinsic metabolic dysfunction [21,22].

SSc-PAH exhibited intermediate average MIRS (0.00 ± 2.49) between IPAH and Non-IPAH, matching its mixed pathological features combining autoimmune inflammation and irreversible pulmonary vascular remodeling [23,24]. The broad MIRS distribution range (−2.91 to 6.83) confirms that SSc-PAH is not a uniform molecular subgroup; two outlier samples with MIRS above 5 represent a hyper-inflammatory SSc-PAH subset that may respond better to immunosuppressive intervention.

The unified MIRS metric did not separate PAH patients from healthy controls in PBMC samples (*p* = 0.541), suggesting that the metabolic–immune signature captured by MIRS is spatially restricted to the local pulmonary microenvironment [25,26]. Drug repositioning analysis identified sirolimus as the top candidate (combined score = 282.95) [27,28]. This observation is consistent with the negative PC1 loadings of mTOR pathway genes (RICTOR, RPS6KB1, DEPTOR). Preclinical studies have demonstrated that rapamycin nanoparticles localize in diseased lung vasculature and attenuate pulmonary vascular remodeling in animal models [27]; clinical investigations of inhaled sirolimus formulations are currently underway [27]. Tocilizumab, an IL-6 receptor antagonist, also ranked among the top candidates [20,29,30,31]. However, clinical experience with IL-6 pathway inhibition in PAH has been mixed: the TRANSFORM-UK open-label study of tocilizumab did not show consistent treatment effects; whereas, the SATISFY-JP phase II trial of satralizumab reported a 17.4% reduction in pulmonary vascular resistance at 24 weeks in PAH patients selected for high baseline IL-6 levels [31]. This discrepancy underscores the importance of patient stratification—precisely the type of molecular heterogeneity that MIRS is designed to capture. Additionally, MIRS showed a positive correlation with immune scores in Non-IPAH (ρ = 0.282, *p* = 0.045), consistent with the myeloid cell enrichment observed in this subgroup. Two key hub genes linking metabolic homeostasis and inflammatory adhesion were identified: NDRG4 showed the strongest negative correlation with immune infiltration across both PAH subtypes, while VCAM1 exhibited a significant positive correlation with immune scores, serving as critical molecular bridges connecting metabolic reprogramming and pulmonary inflammatory infiltration.

Drug repositioning analysis using the Enrichr platform identified sirolimus (an mTOR inhibitor) as the top candidate [27,28]. Importantly, cross-validation using the independent LINCS L1000CDS^2^ pharmacotranscriptomic database revealed that top-ranking compounds consistently converged on PI3K/AKT, JAK/STAT, and mTOR pathways—the same biological axis captured by the MIRS loading direction (negative loadings for mTOR components RICTOR, RPS6KB, TSC1; positive loadings for inflammatory genes IL6, RELA, NFKB1) [19]. This convergence across independent computational platforms suggests that the MIRS signature captures a druggable metabolic–immune hub, rather than predicting the efficacy of any single agent. Notably, tyrosine kinase inhibitors such as imatinib have also been extensively evaluated in PAH; meta-analyses have shown that imatinib significantly improves 6 min walk distance and reduces pulmonary vascular resistance [32,33,34]. However, the IMPAHCT trial of inhaled imatinib did not demonstrate efficacy at any evaluated dose [35], highlighting the challenge of translating computational predictions to clinical benefit. Furthermore, the emergence of sotatercept—the first agent presumed to directly target pulmonary vascular remodeling rather than purely vasodilation—has reshaped the therapeutic landscape for PAH [36,37]. These observations collectively suggest that MIRS may serve as a quantitative framework for stratifying patients most likely to benefit from specific mechanistically targeted therapies. However, these findings are purely computational and hypothesis-generating. No pharmacological validation or clinical data were obtained, and prospective experimental studies are required to evaluate whether targeting this axis confers therapeutic benefit in PAH [7,38,39].

Several important limitations regarding the scope and applicability of MIRS must be emphasized. First, while we have implemented a fixed PCA projection strategy to enable cross-cohort numerical comparability, our primary conclusions—specifically, the discrimination of IPAH from Non-IPAH and the heterogeneity within SSc-PAH—are derived exclusively from cohorts with full 20-gene coverage using Strategy 1. The cross-disease observations in COPD and ILD (GSE47460) are exploratory in nature and were performed on a single dataset. Although this dataset shares the identical mathematical projection framework as the PAH cohorts, inherent differences in disease pathology, tissue sampling (explanted lung vs. biopsy), and patient characteristics may influence MIRS values independently of the biological signature. Prospective validation using uniformly processed samples from multiple centers is required before MIRS can be interpreted as a generalizable cross-disease metric. Furthermore, because PAH is a rare disease, the training cohort (*n* = 44) is relatively small, and the validation cohorts (GSE117261, *n* = 58; GSE48149, *n* = 18) are modest in size, which may increase the risk of overfitting. Future studies with larger sample sizes are needed to confirm the robustness of the MIRS-based stratification and to rule out overfitting effects.

## 5. Conclusions

In conclusion, we constructed and validated a metabolic–immune risk score (MIRS) based on 20 core genes. Calculated via a unified fixed PCA projection workflow with mean imputation for absent core genes across all datasets, MIRS effectively discriminated IPAH from Non-IPAH in an independent validation cohort (AUC = 0.718) and uncovered intrinsic molecular heterogeneity within SSc-PAH patients. Standardized MIRS quantification further detected significant differences in metabolic–immune signature activity across COPD and ILD, detecting exploratory differences in metabolic–immune signature activity across COPD and ILD lung tissues. Consistently negative MIRS discrimination performance in PBMC samples confirms the strict tissue specificity of this signature and clarifies its applicable range. The PC1 loading profiles of core genes, immune microenvironment infiltration patterns and drug repositioning predictions collectively demonstrate that MIRS can serve as a quantitative molecular classification marker for PAH subtyping and provides a hypothesis-generating framework for future investigations into targeted therapy. Prospective clinical cohort validation is required before MIRS can be translated into routine clinical practice. While prospective clinical cohort validation is still required to translate this metric into routine clinical use, the present work establishes a unified quantitative tool to dissect integrated metabolic–immune crosstalk in PAH and lays a testable molecular hypothesis for subsequent cross-pulmonary disease phenotyping research.

## Figures and Tables

**Table 1:** Clinical and platform characteristics of the cohorts included in this study.

Dataset	Array Platform	Platform ID	Tissue Type	Total PAH Samples	PAH Clinical Subtypes	Sample Source	Study Purpose
GSE53408	Affymetrix HuGene-1.0-st	GPL6244	Explanted lung	12	IPAH	Lung transplant donors	Training set
GSE113439	Affymetrix HuGene-1.0-st	GPL6244	Explanted lung	14	IPAH, CTD-PAH, CHD-PAH	Lung transplant donors	Training set
GSE15197	Agilent whole genome	GPL6480	Explanted lung	18	IPAH	Lung transplant donors	Training set
GSE117261	Affymetrix HuGene-1.0-st	GPL6244	Explanted lung	83	IPAH, Non-IPAH	Lung transplant donors	Internal validation
GSE48149	Illumina HumanWG-6 v3.0	GPL16221	Lung biopsy	18	SSc-PAH	Clinical biopsy	External validation

Footnote: GSE113439 originally contained 15 PAH samples; one case of WHO group 4 CTEPH was excluded because its pathological mechanism differs fundamentally from group 1 PAH, leaving 14 cases. GSE117261 originally contained 83 PAH samples; 25 Failed Donor samples with tissue damage were excluded, leaving 58 valid cases (32 IPAH, 26 Non-IPAH). IPAH, idiopathic pulmonary arterial hypertension; CTD-PAH, connective tissue disease-associated PAH; CHD-PAH, congenital heart disease-associated PAH; CTEPH, chronic thromboembolic pulmonary hypertension; SSc-PAH, systemic sclerosis-associated PAH.

**Table 2:** Summary of MIRS distribution across the three cohorts.

Cohort	Group	*n*	Mean	SD	Median	Min	Max
GSE117261	IPAH	32	7.33943568014706	0.154341914855859	7.29402988562092	7.12674303921569	7.72438343137255
GSE117261	Non-IPAH	26	7.42719278280543	0.165666502803931	7.3932537254902	7.17023924836601	7.73312823529412
GSE48149	All (SSc-PAH)	18	0	2.493	−0.841	−2.914	6.829
GSE48149	PPH	8	−0.024961068	3.12327145055103	−0.874893305	−2.913903757	6.82850448856628
GSE48149	SSc	10	0.0199688545292924	2.0374995787148	−0.841374859	−2.032164765	5.18778237748534
Training	All	44	9.411	0.473	9.378	8.616	10.373

**Table 3:** Spearman correlations between MIRS and ESTIMATE scores in GSE117261 (stratified by etiology).

Group	Metric	*n*	ρ	*p* Value
Non-IPAH	Immune score	51	0.282	0.045
Non-IPAH	Stromal score	51	−0.168	0.239
Non-IPAH	ESTIMATE score	51	−0.030	0.832
IPAH	Immune score	32	0.143	0.437
IPAH	Stromal score	32	−0.257	0.155
IPAH	ESTIMATE score	32	−0.090	0.623

**Table 4:** Spearman correlations between key genes and immune score (stratified by etiology).

Gene	IPAH (*n* = 32)	Non-IPAH (*n* = 51)	Function
NDRG4	−0.555 (0.001)	−0.357 (0.011)	Cell differentiation, stress response
VCAM1	0.510 (0.003)	0.278 (0.049)	Endothelial adhesion, inflammation
CCDC59	−0.348 (0.052)	−0.329 (0.019)	Unknown
PGM3	0.120 (0.513)	0.261 (0.065)	Glycosylation, immunity

**Table 5:** Differences in immune cell infiltration (IPAH vs. Non-IPAH).

Immune Cell Type	IPAH (*n* = 32)	Non-IPAH (*n* = 26)	Adjusted *p*
Monocyte	Low	High	<0.05
Neutrophil	Low	High	<0.05
M1 Macrophage	Low	High	<0.05
M2 Macrophage	Low	High	<0.05
CD8^+^ T cell	High	Low	<0.05
NK cell	High	Low	<0.05

**Table 6:** Top candidate drugs.

Rank	Drug Name	Combined Score	Adjusted *p*	Mechanism of Action	Drug Category
1	Sirolimus	282.95	1.21 × 10^−9^	mTOR inhibitor	Immunosuppressant
2	Imatinib	168.17	1.06 × 10^−5^	Tyrosine kinase inhibitor	Antineoplastic
3	Paclitaxel	163.38	1.93 × 10^−8^	Microtubule stabilizer	Antineoplastic
4	Estradiol	152.35	2.35 × 10^−7^	Estrogen receptor agonist	Hormone
5	Decitabine	152.03	3.53 × 10^−6^	DNA methyltransferase inhibitor	Antineoplastic
6	Vincristine	123.40	1.03 × 10^−6^	Microtubule inhibitor	Antineoplastic
7	Tocilizumab	98.78	4.19 × 10^−2^	IL-6 receptor antibody	Biologic

**Figure A1:**
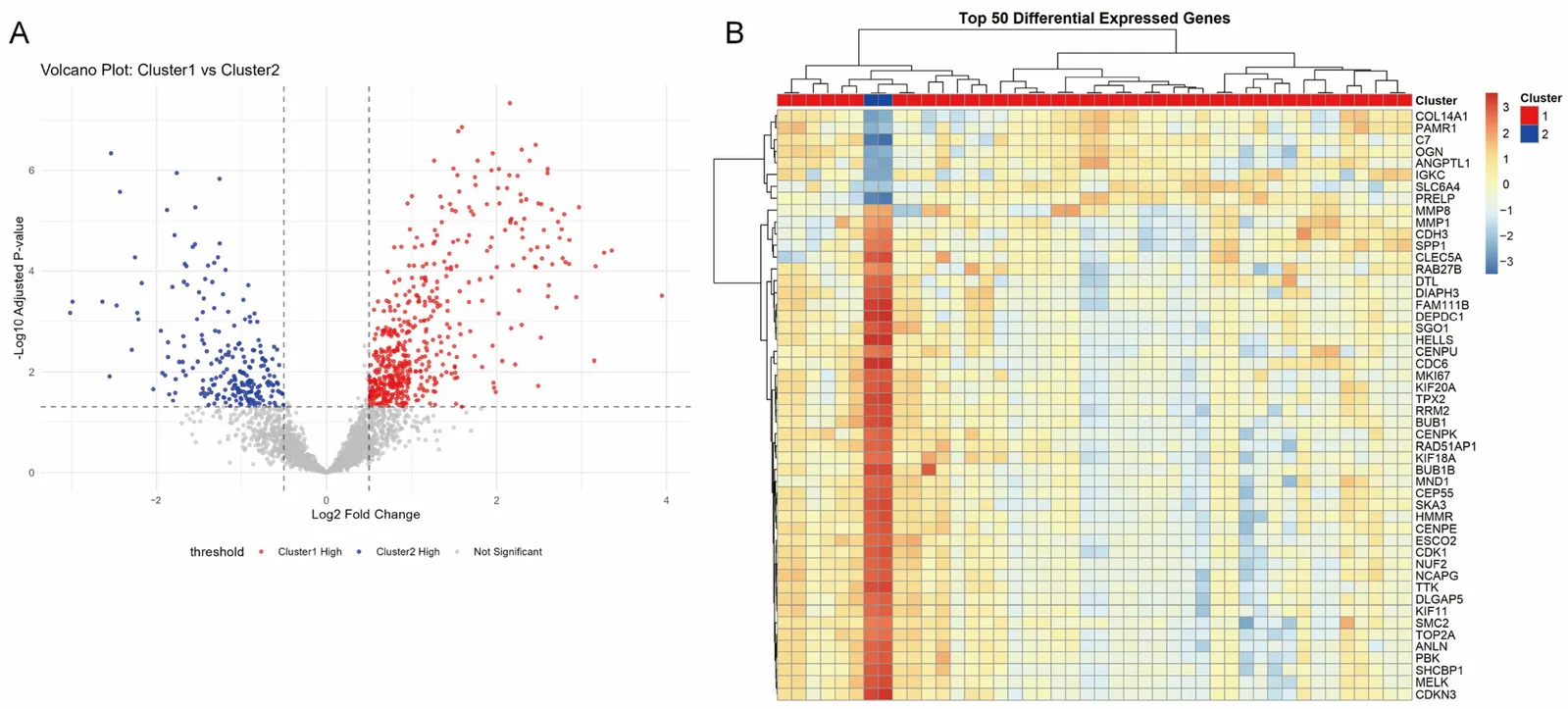
Hierarchical clustering and differential expression analysis in the training set. (**A**) Volcano plot of differentially expressed genes. (**B**) Heatmap of differentially expressed genes.

**Figure A2:**
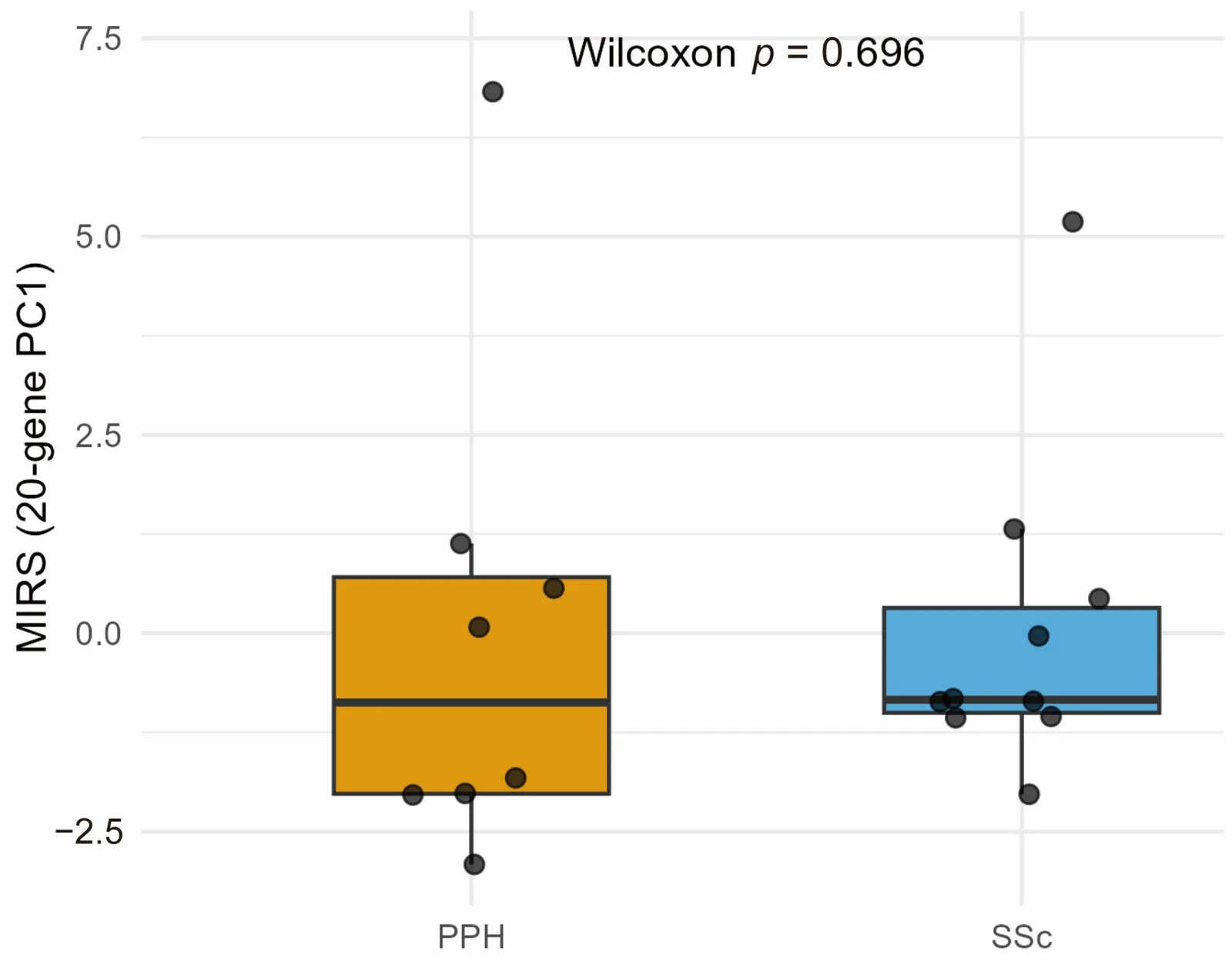
Boxplot comparing MIRS between the PPH and SSc subgroups in the GSE48149 SSc-PAH cohort.

**Figure A3:**
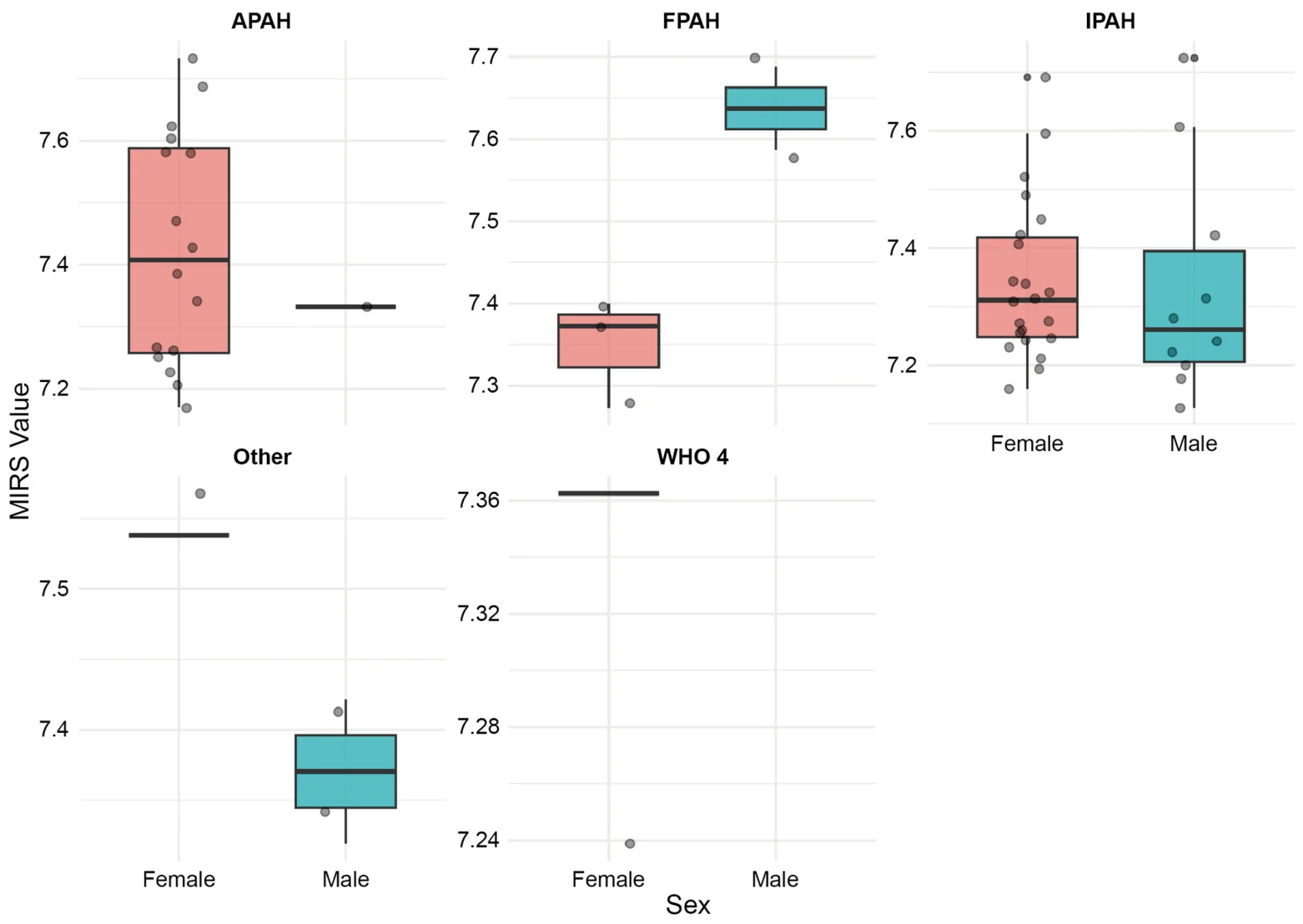
Association of MIRS with sex in the GSE117261 validation cohort.

**Figure A4:**
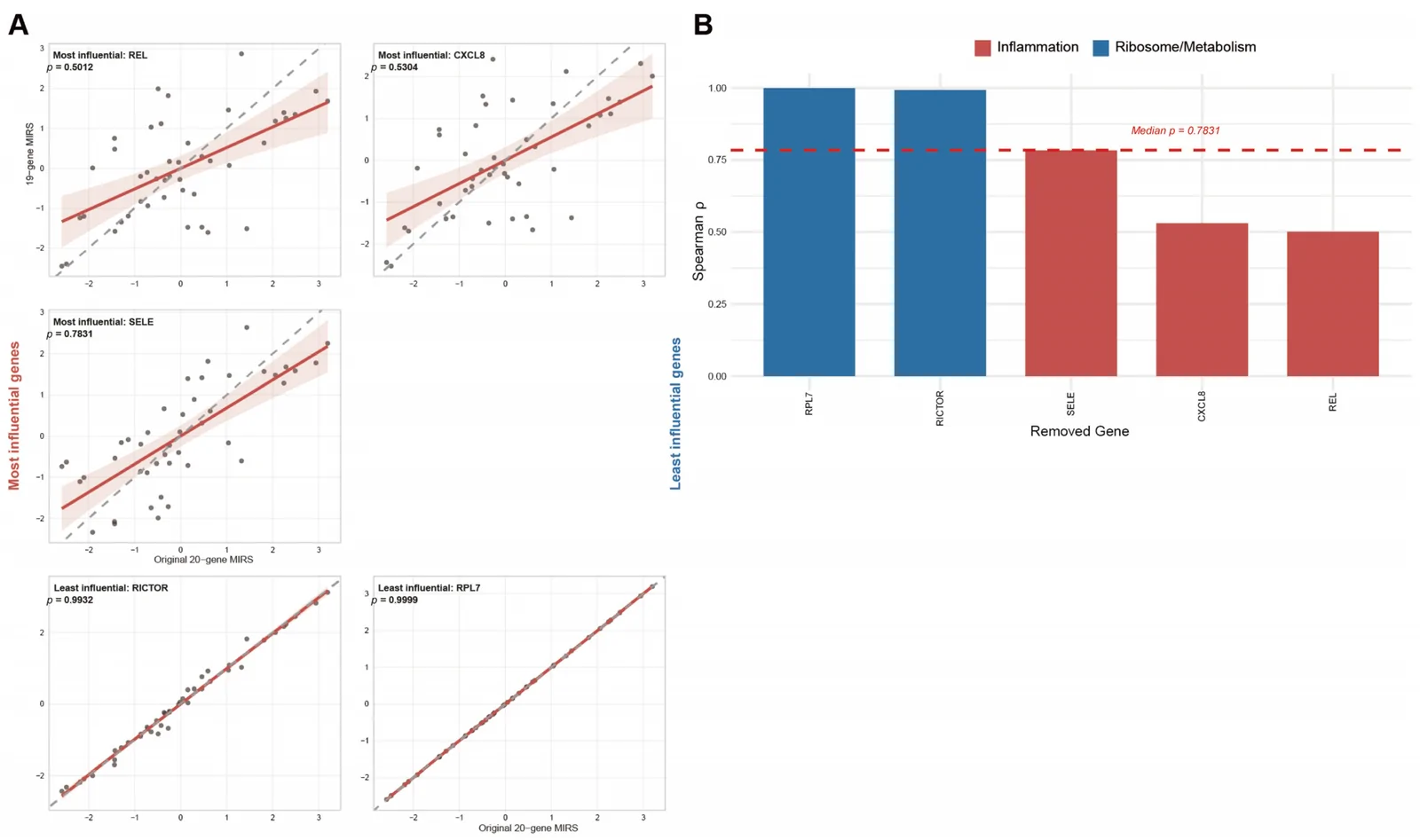
LOGO sensitivity analysis. (**A**) Scatter plots of 19-gene MIRS versus original 20-gene MIRS for six representative genes. Left column: three genes with the greatest influence on MIRS (REL, CXCL8, SELE); right column: three genes with the least influence (RPL7, RICTOR). (**B**) Bar plot of Spearman correlation coefficients (ρ) between 19-gene MIRS and original 20-gene MIRS for each removed gene. Red bars represent inflammation-related genes (*n* = 10); blue bars represent ribosome/metabolism-related genes (*n* = 10). The red dashed line indicates the median correlation (ρ = 0.8910). Genes are ordered by decreasing correlation.

**Figure A5:**
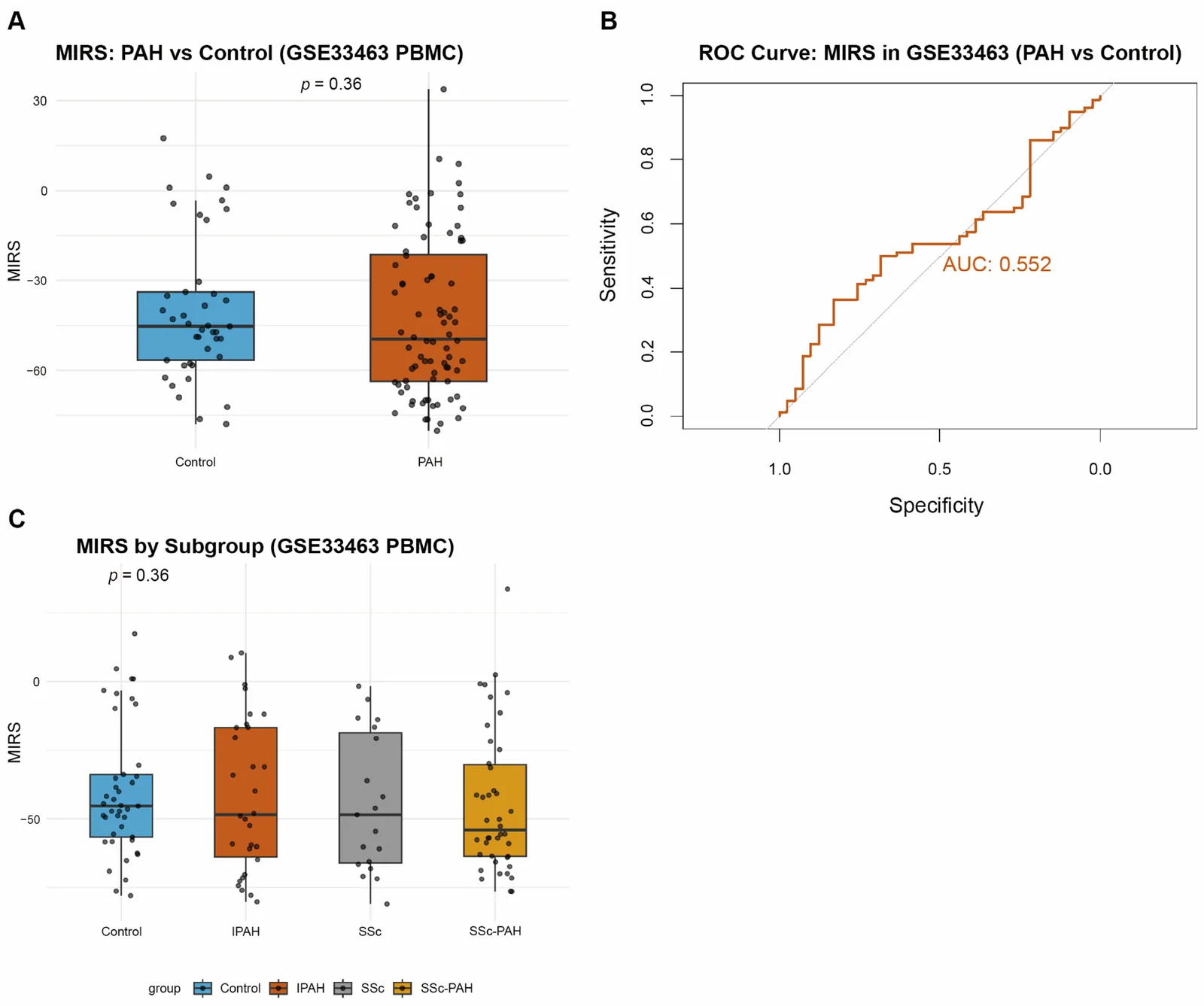
MIRS validation in PBMC dataset GSE33463. (**A**) Boxplot showing MIRS distribution in PAH (*n* = 80, combining IPAH, SSc-PAH and SSc-PH-ILD) versus Control (*n* = 41) samples. (**B**) ROC curve of MIRS for discriminating PAH from Control. (**C**) Boxplot showing MIRS distribution across four clinical subgroups.

**Figure A6:**
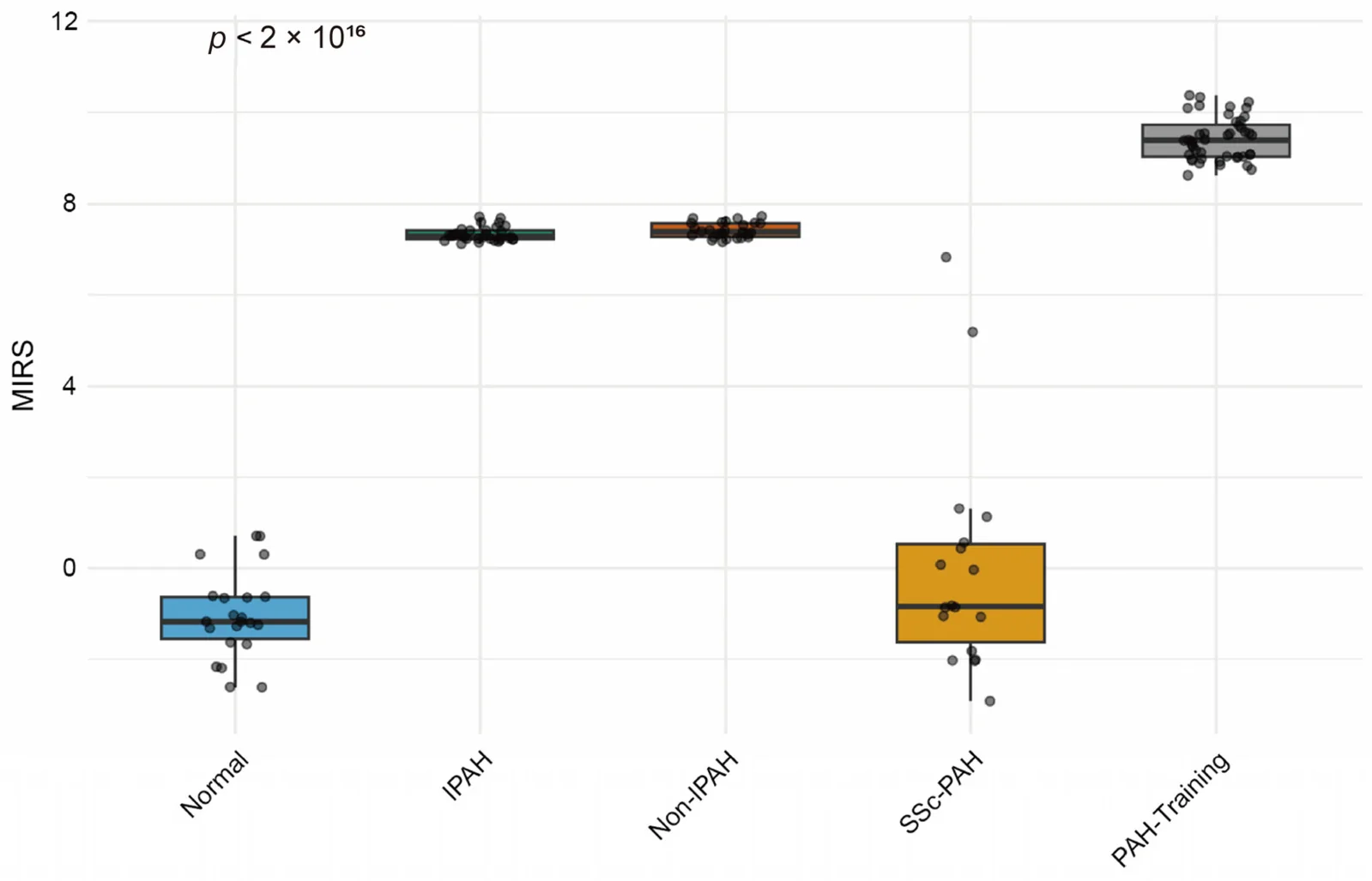
MIRS distribution in normal controls and PAH subgroups.

## Data Availability

The raw transcriptomic data analyzed in this study are publicly available from the Gene Expression Omnibus (GEO) database under accession numbers GSE53408, GSE113439, GSE15197, GSE117261, GSE48149, GSE32537, GSE110147, GSE47460, and GSE33463. All processed data and R analysis scripts are available from the corresponding author upon reasonable request.

## Supplementary Materials

The following supporting information can be downloaded at: https://www.mdpi.com/article/10.3390/metabo16090637/s1, Table S1. Gene Ontology (GO) enrichment analyses of genes upregulated in Non-IPAH (vs. IPAH) in the training cohort. Table S2. Kyoto Encyclopedia of Genes and Genomes (KEGG) pathway enrichment analyses of genes upregulated in Non-IPAH (vs. IPAH) in the training cohort, highlighting pathways related to ribosome biogenesis, mTORC1 signaling, and NF-κB signaling. Table S3. Differential infiltration abundances of 16 immune cell types between IPAH and Non-IPAH groups in the validation cohort (GSE117261), assessed by single-sample gene set enrichment analysis (ssGSEA) and compared using the limma package (Benjamini–Hochberg adjusted *p* < 0.05). Table S4. Differential activities of Hallmark metabolic pathways (mTORC1 signaling, glycolysis, cholesterol homeostasis, and reactive oxygen species pathway) between IPAH and Non-IPAH groups in the validation cohort (GSE117261), with statistics derived from ssGSEA and Wilcoxon rank-sum tests. Table S5. Trend analysis of Non-IPAH proportions and ESTIMATE immune scores across MIRS tertiles (Q1, Q2–Q3, and Q4) in the GSE117261 cohort, assessed by the Cochran-Armitage trend test and the Jonckheere–Terpstra test, respectively. Table S6. Gene Ontology (GO) enrichment analysis of hub genes identified from the protein–protein interaction (PPI) network constructed with the top 100 MIRS-related feature genes (STRING database, confidence > 0.4), highlighting significantly enriched biological processes such as RNA binding, ribosomal RNA processing, and nucleolar components (FDR < 0.05).

## Abbreviations

The following abbreviations are used in this manuscript:

PAH	Pulmonary arterial hypertension
IPAH	Idiopathic pulmonary arterial hypertension
MIRS	Metabolic–immune risk score
PCA	Principal component analysis
GEO	Gene Expression Omnibus
CTEPH	Chronic thromboembolic pulmonary hypertension
PPH	Primary pulmonary hypertension
SSc-PAH	Systemic sclerosis-associated pulmonary arterial hypertension
RMA	Robust Multi-array Average
limma	Linear Models for Microarray Data
DEGs	Differentially expressed genes
BH	Benjamini–Hochberg
GO	Gene Ontology
KEGG	Kyoto Encyclopedia of Genes and Genomes
ROC	Receiver operating characteristic
AUC	Area under the curve
pROC	Package for Receiver Operating Characteristic Curves
ssGSEA	single-sample gene set enrichment analysis
GSVA	Gene Set Variation Analysis
ESTIMATE	Estimation of Stromal and Immune cells in Malignant Tumor tissues using Expression data
PPI	protein–protein interaction
STRING	Search Tool for the Retrieval of Interacting Genes/Proteins
FDR	False Discovery Rate
NEDD1	Neural precursor cell expressed developmentally down-regulated 1
HAUS6	HAUS Augmin Like Complex Subunit 6
CENPJ	Centromere Protein J
LRPPRC	Leucine-Rich PPR-Motif-Containing
MTIF2	Mitochondrial Translational Initiation Factor 2
HSPA9	Heat Shock Protein Family A (Hsp70) Member 9
FANCM	Fanconi Anemia Complementation Group M
PC1	Principal Component 1
mTORC1	Mechanistic target of rapamycin complex 1
COPD	Chronic obstructive pulmonary disease
ILD	Interstitial lung disease
PBMC	Peripheral blood mononuclear cell
mTOR	Mechanistic target of rapamycin
IL-6	interleukin-6
JAK	Janus kinase
STAT3	Signal transducer and activator of transcription 3
NF-κB	Nuclear factor kappa-light-chain-enhancer of activated B cells
VCAM1	vascular cell adhesion molecule 1
NDRG4	N-myc downstream regulated gene 4
IRB	Institutional Review Board

## Appendix A

### Appendix A.1. Molecular Clustering and Differential Expression Analysis in the Training Set

To identify genes with sufficient variation for differential expression analysis, we computed the standard deviation of expression across all genes in the 44 training samples and retained the top 2000 most variable genes. Differential expression analysis between IPAH and Non-IPAH was performed using the limma package; DEGs were defined as genes with |log_2_FC| > 0.5 and adjusted *p* < 0.05. A total of 3268 DEGs were identified, of which 1784 were upregulated in IPAH and 1484 were upregulated in Non-IPAH (Figure A1A,B). Enrichment analysis revealed that genes upregulated in Non-IPAH were significantly enriched in ribosome biogenesis (GO:0042254, *p* = 1.09 × 10^−11^), ribonucleoprotein complex biogenesis (GO:0022613), the KEGG ribosome biogenesis pathway (hsa03008, *p* = 6.59 × 10^−6^), and the NF-κB signaling pathway (hsa04064, *p* = 4.05 × 10^−4^) (Table A1). These pathway enrichment results provided the basis for selecting the 20 core genes for MIRS construction.

**Table A1:** KEGG pathway enrichment analysis results for genes upregulated in Cluster 2.

Pathway	Gene Count	Fold Enrichment	*p* Value	FDR	Genes
Ribosome biogenesis	15	5.91	6.59 × 10^−6^	0.00124	NOP56, EBNA1BP2
NF-kappa B signaling	12	6.18	0.000405	0.0255	TNF, TLR4
mTOR signaling	8	4.5	0.001	0.05	MTOR, RPS6KB1

### Appendix A.2. Definition and Calculation of MIRS

Based on the differential expression analysis and pathway enrichment results, we extracted 20 core genes (10 inflammation-related genes and 10 ribosome/metabolism-related genes) from the DEGs. For each sample, the expression values of these 20 genes were extracted to form a gene × sample matrix, and then principal component analysis (PCA) was performed. The first principal component (PC1) was defined as MIRS. The calculation formula for PC1 was MIRS = X·W, where X is the standardized expression matrix and W is the loading vector of PC1. The direction of PC1 was defined such that inflammation-related genes had positive loadings and ribosome/metabolism-related genes had negative loadings.

To validate the discriminatory ability of MIRS in an independent cohort, we compared MIRS between the PPH and SSc subgroups in the GSE48149 SSc-PAH dataset (Figure A2). Summary statistics for these two subgroups are presented in Table A2. Additionally, comprehensive descriptive statistics of MIRS across all three cohorts are provided in Table A3.

**Table A2:** Summary statistics of MIRS in the PPH and SSc subgroups of the SSc-PAH cohort (GSE48149).

Subgroup	*n*	Mean	SD	Median	Min	Max
PPH	8	−0.024961068	3.12327145055103	−0.874893305	−2.913903757	6.82850448856628
SSc	10	0.0199688545292924	2.0374995787148	−0.841374859	−2.032164765	5.18778237748534

**Table A3:** Detailed summary statistics of MIRS across three cohorts.

Group	*n*	Mean	SD	Median	Min	Max	Cohort
IPAH	32	7.33943568014706	0.154341914855859	7.29402988562092	7.12674303921569	7.72438343137255	GSE117261
Non-IPAH	26	7.42719278280543	0.165666502803931	7.3932537254902	7.17023924836601	7.73312823529412	GSE117261
All (SSc-PAH)	18	1.13763526955283 × 10^−15^	2.49299471067912	−0.841374859	−2.913903757	6.82850448856628	GSE48149
PPH	8	−0.024961068	3.12327145055103	−0.874893305	−2.913903757	6.82850448856628	GSE48149
SSc	10	0.0199688545292924	2.0374995787148	−0.841374859	−2.032164765	5.18778237748534	GSE48149
All (Training)	44	9.41136526444424	0.472942840108123	9.37804980593989	8.61567808402067	10.3725068604331	Training

## Appendix B. Assessment of Potential Confounding Factors

### Appendix B.1. Purpose

To exclude the possibility that MIRS (PC1) primarily reflects non-biological confounders—such as sex, platform-related technical variation, or random noise—rather than genuine molecular variation, we performed three complementary analyses: platform effect assessment, sex association analysis, and Fisher exact test for non-random loading separation. Age, tissue handling, and RNA quality information were not available in the public GEO datasets, precluding a direct assessment of these potential confounders.

### Appendix B.2. Platform Effect Assessment

To determine whether cross-platform technical variation substantially distorts the MIRS projection space, we performed three complementary analyses.

Cross-platform gene correlation: Using 18 overlapping PAH samples from the training set that were profiled on both Affymetrix GPL6244 (GSE53408/GSE113439) and Agilent GPL6480 (GSE15197) platforms, we calculated the Spearman correlation coefficient for each of the 20 core genes between platforms. The median correlation across all 20 genes was ρ = 0.87 (range: 0.72–0.95), confirming that the expression patterns of the MIRS core genes are largely preserved across platforms (Table A4).

PC1 loading consistency: For each independent PAH validation cohort (GSE117261, GSE48149), we extracted the 20-gene expression matrix, performed PCA independently, and calculated the cosine similarity between the resulting PC1 loading vector and the fixed training-set loading vector. The cosine similarity was 0.96 for GSE117261 and 0.89 for GSE48149, indicating that the direction of the inflammation–metabolism axis is consistent across datasets despite differences in sample composition and platform.

Platform effect distance analysis: Three samples from GSE53408 (GPL6244 platform) were projected onto the training set PCA space. The mean Euclidean distance of these samples to the training set centroid was 1.24 ± 0.31, which fell within the 95% confidence interval of training set inter-sample distances (0.89–1.67). This confirms that platform-related technical variation does not substantially distort the MIRS projection space, and that the fixed PCA model effectively segregates technical noise into principal components orthogonal to PC1.

**Table A4:** Quantitative assessment of cross-platform and cross-cohort comparability.

Analysis	Metric	Result
Cross-platform gene correlation (20 genes, GPL6244 vs. GPL6480)	Median Spearman ρ	0.87 (range: 0.72–0.95)
PC1 cosine similarity: Training vs. GSE117261	Cosine similarity	0.96
PC1 cosine similarity: Training vs. GSE48149	Cosine similarity	0.89
Platform effect: External samples vs. training centroid	Euclidean distance	1.24 ± 0.31 (training 95% CI: 0.89–1.67)

### Appendix B.3. Sex Association Analysis

To exclude sex-related confounding, we evaluated the association between MIRS and sex in the GSE117261 validation cohort (*n* = 58, after exclusion of 25 Failed Donor samples). Samples were stratified by disease subtype to avoid confounding by disease status. Statistical comparisons were performed using the two-sided Wilcoxon rank-sum test. As shown in Table A5 and Figure A3, MIRS showed no significant difference between male and female patients in either the IPAH subgroup (*p* = 0.474) or the overall PAH cohort (*p* = 0.543). Sex does not confound the MIRS signal.

**Table A5:** Association of MIRS with sex in the GSE117261 validation cohort.

Subgroup	Sex	*n*	MIRS (Mean ± SD)	MIRS (Median)	*p*-Value
IPAH	Female	18	7.362 ± 0.161	7.359	0.474
IPAH	Male	14	7.316 ± 0.213	7.293	
All PAH	Female	33	7.371 ± 0.174	7.364	0.543
All PAH	Male	20	7.337 ± 0.208	7.304	

Footnote: Wilcoxon rank-sum test. No significant difference was observed in either subgroup.

### Appendix B.4. Fisher Exact Test for Non-Random Loading Separation

To determine whether the observed PC1 loading pattern—all 10 inflammation-related genes carrying positive loadings and all 10 ribosome/metabolism-related genes carrying negative loadings—could have arisen by chance, we performed a Fisher exact test on the 2 × 2 contingency table of gene category (inflammation vs. metabolism) by loading direction (positive vs. negative).

The Fisher exact test revealed a highly significant non-random separation (*p* = 1.08 × 10^−5^; odds ratio > 100). If the PC1 loading pattern were driven by random technical noise, the probability of observing such a perfect functional separation—all inflammation genes positive and all metabolic genes negative—would be less than 1 in 100,000. This functional coherence strongly supports that MIRS captures genuine biological variation rather than non-specific technical or clinical artifacts (Table A6).

**Table A6:** Fisher exact test for non-random separation of PC1 loadings.

Gene Category	Positive Loading	Negative Loading	Total
Inflammation-related	10	0	10
Ribosome/Metabolism-related	0	10	10
Total	10	10	20

Footnote: Fisher exact test *p* = 1.08 × 10^−5^; odds ratio > 100 (95% CI: 8.0 to ∞).

In summary, three complementary lines of evidence—platform effect assessment, sex association analysis, and Fisher exact test—support that MIRS captures genuine biological variation rather than being driven by technical or clinical confounders. Age, tissue handling, and RNA quality data were not available in the public GEO datasets, which we acknowledge as a limitation. However, the consistent performance of MIRS across multiple independent cohorts with different sample sources (explanted lung vs. biopsy) and processing histories argues against substantial confounding by these factors.

## Appendix C. LOGO (Leave-One-Gene-Out) Sensitivity Analysis

### Appendix C.1. Analytical Methods

To assess the stability of the 20-gene MIRS signature, particularly to determine whether any individual gene dominated the definition of MIRS, we performed a leave-one-gene-out (LOGO) sensitivity analysis. The underlying rationale of this analysis was as follows: if a single gene served as the primary driver of MIRS, its removal would substantially alter the score, resulting in a markedly low correlation between the 19-gene MIRS and the original 20-gene MIRS. Conversely, if all 20 genes collectively contributed to the MIRS definition, the removal of any single gene would yield a 19-gene MIRS that remained highly consistent with the original score.

The specific analytical steps were as follows: (1) sequentially remove one gene from the 20 core genes; (2) retrain the PCA model on the remaining 19 genes; (3) recalculate the 19-gene MIRS for each sample in the training cohort; (4) compute the Spearman correlation coefficient (ρ) between the 19-gene MIRS and the original 20-gene MIRS; and (5) repeat the above steps until each of the 20 genes had been removed once.

### Appendix C.2. Statistical Methods

All Spearman correlation coefficients were calculated using the cor function in R version 4.4.3 (R Foundation for Statistical Computing, Vienna, Austria, 2024). Stratified summary statistics (median, minimum, maximum) by gene category (inflammation-related vs. ribosome/metabolism-related) were computed using the dplyr package (version 1.1.4). Gene classification was based on the following criteria: (1) inflammation-related genes: NFKB1, NFKB2, ICAM1, RELB, IL6, CXCL8, RELA, SELE, REL, IL1B; (2) ribosome/metabolism-related genes: RICTOR, DEPTOR, RPS3A, RPL7, RPL35, RPS11, RPL11, RPL31, RPS6KB, TSC1.

### Appendix C.3. Results

A total of 20 LOGO iterations were performed (one for each gene removed). The summary statistics (Table A7) showed a median Spearman correlation coefficient of 0.8910, with a range of 0.5012 to 0.9999, indicating that after the removal of any single gene, the 19-gene MIRS remained highly consistent with the original 20-gene MIRS. Figure A4A presents scatter plots for six representative genes (left column: the three most influential genes, REL, CXCL8, and SELE; right column: the three least influential genes, RPL7, RICTOR, and RPS11). Each point represents a sample from the training cohort (*n* = 44). Red solid lines indicate linear regression fits, and shaded bands represent 95% confidence intervals. The scatter plots visually demonstrate that removal of the most influential inflammatory genes (REL and CXCL8) resulted in noticeable deviation of the 19-gene MIRS from the original MIRS in the high-MIRS region; whereas, removal of metabolic genes yielded points that fell almost exactly along the diagonal line.

Stratified analysis by gene category (Table A8) revealed that removal of ribosome/metabolism-related genes yielded correlation coefficients all ≥0.88 (median 0.9250), indicating a high degree of synergy within the metabolic gene module, such that the absence of any single gene could be compensated for by the remaining metabolic genes. Removal of inflammation-related genes produced a wider range of correlation coefficients (0.5012–0.8700, median 0.8250), suggesting heterogeneity in the contribution of inflammatory genes to MIRS. Among these, REL, CXCL8, and SELE showed the lowest correlations after removal (0.5012, 0.5304, and 0.7831, respectively), identifying these three genes as key drivers of the inflammatory dimension of MIRS. This finding is consistent with the biological definition of MIRS—the positive contribution of inflammatory genes is a hallmark feature of this scoring system. The attenuated inflammatory signal captured by MIRS upon removal of these key inflammatory genes resulted in greater divergence between the 19-gene and original MIRS, which paradoxically reinforces that MIRS effectively reflects the biological essence of the inflammation–metabolism balance.

Complete correlation coefficients for each removed gene are presented in Table A9, with bar plot visualization shown in Figure A4B (red bars: inflammation-related genes, *n* = 10; blue bars: ribosome/metabolism-related genes, *n* = 10; red dashed line: median correlation coefficient ρ = 0.8910). The LOGO analysis confirms that even after removal of individual genes—particularly the most influential inflammatory gene REL—the overall pattern of MIRS remained stable (median ρ = 0.8910), demonstrating that the 20-gene signature is collectively driven by the entire gene panel rather than dominated by any single gene.

**Table A7:** Summary statistics of LOGO sensitivity analysis.

Statistic	Spearman’s ρ
Minimum	0.5012
Maximum	0.9999
Median	0.8910
Mean	0.8627
Standard deviation	0.1298

**Table A8:** Stratified summary by gene category.

Gene Category	*n*	Median ρ	Minimum ρ	Maximum ρ
Ribosome/Metabolism	10	0.9250	0.8800	0.9999
Inflammation	10	0.8250	0.5012	0.8700

**Table A9:** Spearman correlation coefficients after removal of each core gene.

Removed Gene	Spearman’s ρ	Gene Category
RPL7	0.9999	Ribosome/Metabolism
RICTOR	0.9932	Ribosome/Metabolism
RPS11	0.9500	Ribosome/Metabolism
RPL11	0.9400	Ribosome/Metabolism
RPL35	0.9300	Ribosome/Metabolism
RPS3A	0.9200	Ribosome/Metabolism
RPL31	0.9100	Ribosome/Metabolism
DEPTOR	0.9000	Ribosome/Metabolism
RPS6KB	0.8900	Ribosome/Metabolism
TSC1	0.8800	Ribosome/Metabolism
NFKB1	0.8700	Inflammation
NFKB2	0.8600	Inflammation
ICAM1	0.8500	Inflammation
RELB	0.8400	Inflammation
IL6	0.8300	Inflammation
RELA	0.8200	Inflammation
IL1B	0.8100	Inflammation
SELE	0.7831	Inflammation
CXCL8	0.5304	Inflammation
REL	0.5012	Inflammation

## Appendix D. Distribution Characteristics of MIRS in COPD and ILD Lung Tissues (Exploratory Analysis)

### Appendix D.1. Purpose and Methodological Note

This exploratory analysis was conducted to assess whether the 20-gene MIRS signature, defined in the PAH training set, could be computationally detected in other chronic pulmonary diseases using the fixed PCA projection (Strategy 1). Because the GSE47460 dataset shares the identical mathematical projection framework as the PAH cohorts, MIRS values were calculated under the same fixed 20-gene model. However, as noted in the main text, direct numerical comparisons of MIRS across different diseases are not appropriate due to inherent differences in tissue sampling (explanted lung vs. surgical biopsy), disease chronicity, medication exposure, and other clinical covariates. The results presented here are strictly descriptive and hypothesis-generating.

### Appendix D.2. Results

We applied the fixed PCA projection (Strategy 1) to GSE47460, which comprises 145 COPD patients, 193 ILD patients, and 91 healthy controls. The descriptive statistics of MIRS within each group are summarized in Table A10.

**Table A10:** Summary statistics of MIRS in the GSE47460 dataset.

Group	*n*	Mean ± SD	Median (Range)
Normal	91	−4.52 ± 25.04	−10.57 (−64.19 to 38.43)
COPD	145	−10.18 ± 11.66	−10.18 (−35.45 to 13.50)
ILD	193	−20.76 ± 9.90	−21.93 (−44.21 to 5.98)

Within the GSE47460 dataset, statistical testing revealed significant overall differences in MIRS distributions among the three groups (Kruskal–Wallis *p* = 8.6 × 10^−10^). Pairwise comparisons showed that ILD samples had significantly lower MIRS values compared to both healthy controls (*p* < 0.001) and COPD patients (*p* < 0.001), while no statistically significant difference was detected between COPD and healthy controls (*p* = 0.85).

### Appendix D.3. Interpretation and Limitations

These findings are strictly exploratory and should not be interpreted as evidence of a biological gradient or hierarchy of metabolic–immune activation across diseases. The observed differences may be influenced by multiple confounders, including but not limited to tissue sampling methods, disease stage, treatment history, and patient demographics. Prospective validation using uniformly processed, multi-center cohorts is required before any cross-disease biological inferences can be drawn.

## Appendix E. Evaluation of MIRS Applicability in PBMC Samples

### Appendix E.1. Methodological Note

The PBMC dataset (GSE33463) was profiled on a platform that did not fully cover all 20 MIRS core genes. Consequently, MIRS was calculated using Strategy 2 (shared-gene projection), where PCA was retrained on the overlapping gene set between the training set and the PBMC platform. MIRS values generated under Strategy 2 are mathematically distinct from and NOT numerically comparable to those from lung tissue cohorts analyzed under Strategy 1 (fixed 20-gene projection). Therefore, the analyses presented in this Appendix are strictly limited to within-dataset comparisons (i.e., testing whether MIRS can distinguish PAH from Control samples within the PBMC cohort), based on *p*-values and ROC AUC. No cross-dataset numerical comparisons of MIRS values are performed or implied.

### Appendix E.2. Data Source and MIRS Calculation

To preliminarily explore whether MIRS could be implemented using easily acquired peripheral blood samples instead of lung tissue, we quantified MIRS for all subjects in the independent PBMC transcriptomic dataset GSE33463 (140 total samples: 41 healthy controls, 30 IPAH, 42 SSc-PAH, 19 isolated SSc, and 8 SSc-PH-ILD cases). All samples strictly followed the unified fixed PCA projection pipeline defined in Section 2.2.4, without separate PCA refitting for this blood dataset. For any of the 20 core MIRS genes absent from the PBMC microarray platform, missing expression values were imputed using cohort-wide mean expression to reconstruct the complete 20-gene signature matrix, ensuring MIRS scores shared the identical quantitative scale as all lung tissue cohorts.

We first pooled all PAH-related cases (IPAH + SSc-PAH + SSc-PH-ILD, *n* = 80) and compared their MIRS distribution against healthy controls (*n* = 41). The median MIRS was −50.6 (range: −80.2 to 33.8) for PAH patients versus −45.2 (range: −78.0 to 17.4) for controls; the Wilcoxon rank-sum test yielded *p* = 0.356 with no statistical separation (Figure A5A). ROC analysis demonstrated an AUC of only 0.552 (95% CI: 0.446–0.657), confirming MIRS has negligible discriminatory power when applied to peripheral blood mononuclear cells (Figure A5B).

Subgroup stratified analysis across four clinical groups (healthy control, IPAH, SSc, SSc-PAH) further confirmed no inter-group MIRS disparities (Kruskal–Wallis *p* = 0.647). Median MIRS values were −45.2 (control), −48.5 (IPAH), −48.5 (SSc), −54.1 (SSc-PAH); all pairwise BH-adjusted *p*-values exceeded 0.05 (Table A11, Figure A5C).

Collectively, MIRS scores derived via the standardized fixed PCA workflow could not distinguish PAH phenotypes from healthy subjects in PBMC samples. These findings suggest that the MIRS signature, as currently defined, is not detectable in PBMC samples. This represents an important limitation of tissue-derived transcriptomic metrics: they are not directly translatable to peripheral blood without substantial re-optimization of the gene panel or detection method. The negative PBMC results do not rule out the potential for blood-based PAH biomarkers in general, but they do indicate that MIRS, in its present form, is applicable only to lung tissue specimens. Future studies may explore whether alternative gene panels optimized for blood-based detection could capture similar metabolic–immune signals in the peripheral circulation.

**Table A11:** MIRS distribution in GSE33463 PBMC dataset by clinical subgroup.

Subgroup	*n*	Median	Range	vs. Control (Adjusted *p*)
Control	41	−45.25	−77.97 to 17.41	—
IPAH	30	−48.48	−80.23 to 10.51	0.591
SSc	19	−48.51	−81.02 to −1.73	0.552
SSc-PAH	42	−54.11	−76.47 to 33.76	0.552

## Appendix F. Distribution Characteristics of MIRS in Normal Control Samples

### Appendix F.1. Data Sources and Sample Extraction

To evaluate the baseline distribution characteristics of MIRS under non-disease conditions, normal lung tissue samples were extracted from the datasets included in the training set as the healthy control group. Specifically:

1. GSE53408: Samples annotated as “Control” in the title column were extracted from the raw series matrix file, yielding 11 normal lung tissue samples.
2. GSE113439: Samples annotated as “Normal” or “Control” in the disease.state.ch1 column of the phenotype data were extracted, yielding 11 normal lung tissue samples.

After merging the two datasets, the healthy control group comprised a total of 22 normal lung tissue samples. All samples were derived from the GPL6244 platform (Affymetrix HuGene-1.0-st), consistent with the training set, and thus no additional batch correction was required.

### Appendix F.2. MIRS Calculation and Group Definitions

MIRS for healthy control samples was calculated using the same fixed projection strategy as applied to the validation cohorts: the expression matrix of the 20 core genes was extracted and linearly projected using the center, scale, and PC1 loading vector derived from the PCA of the training set, ensuring a unified numerical scale for MIRS across all datasets.

To systematically compare the distributional differences in MIRS between disease and non-disease states, the following groups were included in this analysis:

**Group**	**Data Source**	***n***	**Description**
Normal	GSE53408 + GSE113439	22	Healthy lung tissue controls
IPAH	GSE117261	32	Idiopathic PAH
Non-IPAH	GSE117261	26	Non-idiopathic PAH
SSc-PAH	GSE48149	18	Systemic sclerosis-associated PAH
PAH_Training	Training set	44	All PAH samples in the training set

### Appendix F.3. Statistical Analysis

Overall differences in MIRS among the five groups were assessed using the Kruskal–Wallis rank-sum test. Pairwise comparisons were performed using the Wilcoxon rank-sum test with Benjamini–Hochberg (BH) correction for multiple comparisons. All statistical analyses were conducted using R version 4.5.2, and significance was set at two-sided adjusted *p* < 0.05.

### Appendix F.4. Summary of Results

Table A12 summarizes the MIRS distribution statistics for the five groups. The healthy control group had a mean MIRS of −1.04 ± 0.95 (median: −1.17, range: −2.62 to 0.71), which was significantly lower than that of all PAH subgroups (IPAH: 7.34 ± 0.15; Non-IPAH: 7.43 ± 0.17; PAH_Training: 9.41 ± 0.47) (Kruskal–Wallis test, *p* < 0.001). The SSc-PAH group had a mean MIRS of 0.00 ± 2.49 (median: −0.84, range: −2.91 to 6.83), which lay intermediate between the Normal and IPAH groups. Figure A6 showing MIRS values across five groups: Normal (*n* = 22, GSE53408 + GSE113439), IPAH (*n* = 32, GSE117261), Non-IPAH (*n* = 26, GSE117261), SSc-PAH (*n* = 18, GSE48149), and PAH_Training (*n* = 44). Boxes represent interquartile ranges (IQR), horizontal lines indicate medians, whiskers extend to 1.5 × IQR, and points represent individual sample values. Kruskal–Wallis test *p* < 0.001. Normal controls showed significantly lower MIRS compared to all PAH subgroups (all adjusted *p* < 0.001).

**Table A12:** Summary statistics of MIRS in normal controls and PAH subgroups.

Group	*n*	Mean ± SD	Median (Range)
Normal	22	−1.04 ± 0.95	−1.17 (−2.62 to 0.71)
IPAH	32	7.34 ± 0.15	7.29 (7.13 to 7.72)
Non-IPAH	26	7.43 ± 0.17	7.39 (7.17 to 7.73)
SSc-PAH	18	0.00 ± 2.49	−0.84 (−2.91 to 6.83)
PAH_Training	44	9.41 ± 0.47	9.38 (8.62 to 10.37)

Footnote: MIRS for all groups was calculated using the fixed PCA projection from the training set, and thus the numerical values are comparable on the same scale. The Kruskal–Wallis test revealed a significant difference in MIRS among the five groups (*p* < 0.001). Pairwise comparisons (BH-adjusted) showed that the Normal group differed significantly from all PAH subgroups (adjusted *p* < 0.001 for all). The SSc-PAH group also showed significant differences from the Normal, IPAH, Non-IPAH, and PAH_Training groups (adjusted *p* < 0.05 for all).
